# Supplementary material for: Skin-inspired, sensory robots for electronic implants
Source: Nat Commun. 2024 Jun 5;15:4777. doi: 10.1038/s41467-024-48903-z (PMC11153219; doi:10.1038/s41467-024-48903-z)
Supplement: Supplementary file 1 — Supplementary Information [file 41467_2024_48903_MOESM1_ESM.pdf]

Skin-inspired, sensory robots for electronic implants

Lin Zhang[1], Sicheng Xing[2], Haifeng Yin[3], Hannah Weisbecker[4], Hiep Thanh Tran[2], Ziheng Guo[5], Tianhong Han[6], Yihang Wang[1], Yihan Liu[1], Yizhang Wu[1], Wanrong Xie[1], Chuqi Huang[1], Wei Luo[2], Michael Demaesschalck[5], Collin McKinney[5], Samuel Hankley[5], Amber Huang[4], Brynn Brusseau[4], Jett Messenger[7], Yici Zou[4], Wubin Bai[1*]

[1]Department of Applied Physical Sciences, University of North Carolina, Chapel Hill, NC 27514, USA.

[2]Department of Biomedical Engineering, University of North Carolina, Chapel Hill, NC 27514, USA.

[3]MCAllister Heart Institute Core, University of North Carolina, Chapel Hill, NC 27514, USA.

[4]Department of Biology, University of North Carolina, Chapel Hill, NC 27514, USA.

[5]Department of Chemistry, University of North Carolina, Chapel Hill, NC 27514, USA.

[6]Joint Department of Biomedical Engineering, North Carolina State University, Raleigh, NC 27606, USA.

[7]Weldon School of Biomedical Engineering, Purdue University, West Lafayette, IN 47907, USA.

*To whom correspondence should be addressed to wbai@unc.edu (W.B.)

This Supplementary Information includes:

Supplementary Note S1: The XPS analysis of functional nanocomposites

Supplementary Note S2: The XRD and FTIR analysis of functional nanocomposites

Supplementary Note S3: The sensory-motor integration within the soft robotic system

Supplementary Note S4: Wireless sensing and actuation of a soft sensory robot

Supplementary Note S5: The effect of size and orientation of the coil on the wireless power transfer

Supplementary Note S6: Application-oriented device customization

Supplementary Note S7: *In vivo* animal test of a soft robotic thera-gripper

Table S1 Parameter comparison of different hydrogel-based actuators

Table S2 Aspect ratio comparison of different minimally invasive implants

Figs. S1 to S52

Legend for Movie 1-6

Supplementary Note S1: The XPS analysis of functional nanocomposites

Fig. S12 shows the XPS spectra of the RGO/PI nanocomposite. The C1s spectrum can be separated into two components C-C (285.4 eV) and C=O (287.4 eV), respectively (Fig. S12A). As shown in Fig. S12B, the peak at 400.1 eV in the N1s spectrum arises from -CONH, indicating that the RGO sheets are “welded” by PI through the formation of -CONH bindings between terminal amine groups of PI and residual –COOH or C–O–C groups in RGO. The peak at 532.4 eV in the O1s spectrum is due to the C-O groups in the composite (Fig. S12C) [55]. Fig. S13 shows the XPS spectra of the AgNWs/PI nanocomposite. The C1s peak at 284.9 eV in Fig. S13A is identified as a C-O bond, while peak at 287.9 eV belongs to O=C-O bond. The N1s peak at 400.0 eV is related to the pyridinic nitrogen of PVP (Fig. S13B). In the O1s spectrum, the carbonyl group was detected at 531.3 eV. The peak at 532.8 eV indicates the surfaces of AgNWs are strongly coordinated with O atoms in PI (Fig. S13B). The spectrum of Ag3d contains two peaks at 374.2 eV and 368.2 eV, corresponding to 3d3/2 peaks and 3d5/2 peaks of metallic silver, respectively (**Fig. S13D**) [58,59]. **Fig. S14** exhibits the XPS spectra of the PEDOT:PSS/PI nanocomposite. C1s spectrum is demonstrated in **Fig. S14A**. The peak of aromatic C=C of PEDOT is at 284.6 eV. The C-O/C-S and C=O/C=S bonds are at 286.7 eV and 288.7 eV, respectively. **Fig. S14B** is the N1s spectrum, in which the peak at 400.1 eV is due to N atoms in C-N-H. In the O1s spectrum, the peaks at 531.4 eV and 533.3 eV correspond to S-O and C-O-H, respectively (**Fig. S14C**). In the S2p spectrum, the peaks between 163 eV and 165 eV are assigned to the signal from sulfur atoms of PEDOT. While the peak at 167.6 eV is attributed to sulfur atoms of PSS (**Fig. S14D**) [56,57].

Supplementary Note S2: The XRD and FTIR analysis of functional nanocomposites

Fig. S17G and **Fig. S21D** show the FTIR spectra of the AgNW/PI nanocomposite and RGO/PI nanocomposite, respectively. Characteristic absorption bands of polyimide appear at 1781 cm^{-1}, 1716 cm^{-1} and 1396 cm^{-1}, corresponding to C=O asymmetrical stretching, C=O symmetrical stretching, and C-N stretching, respectively [66]. The AgNW/PI (RGO/PI) spectrum is essentially the direct spectra convolution of AgNWs (RGO) and PI. **Fig. S17H** shows the XRD patterns of AgNW/PI nanocomposite. The peaks at 2θ = 38.24°, 44.42°, 64.61°, 77.45° and 81.55° correspond to the (111), (200), (220), (311) and (222) diffractions from the crystalline Ag [64,65]. **Fig. S21E** shows the XRD patterns of RGO/PI nanocomposite. The peaks at 2θ = 26.47° and 12.52° are assigned to the (002) and (001) diffractions from graphene oxide [65].

Supplementary Note S3: The sensory-motor integration within the soft robotic system

The integrated design that leverages on the synergistic interplay between sensors and soft robotic actuators enables volatile adaptivity and responsiveness of the robotic implant in ever-changing conditions. This design allows sensory inputs to directly influence actuator outputs in real-time, creating a feedback loop that allows for automatic adjustment based on environmental stimuli. This feature underscores our system's responsiveness and adaptability, allowing with the principles of sensor-actuator integration.

As an example, we present an adaptively controlled responsive robotic gripper that maintains optimal actuation temperatures responsive to external temperature changes. The responsive self-adaptation in different thermal environments ensures optimal and predictable heating patterns regardless of conditions, as well as increasing patient safety and preventing potential burn hazard caused by overheating. **Fig. S25A** shows an integrative system that provides temperature sensory readout and, through feedback-controlled power delivery, allows actuation responsive to external thermal environment. **Fig. S25B** demonstrates the working principles of controlling hardware and software. The resistive temperature sensor was connected in series with a reference resistor. The voltage drops over the sensor is read and converted by the on-chip ADC and is processed by the microcontroller to convert to a temperature value. Upon receipt of the temperature value, the control algorithm compares it with the target temperature to produce an error value. The error value is multiplied with a predefined coefficient to produce a corrective factor and is added to the current output value to generate a new output value. The on-chip pulse-width modulation (PWM) module converts the output value to a PWM voltage signal, which is then amplified by the on-board power MOSFET, forming a controller current source to produce a current through the heater. When the external temperature changes, a large corrective factor is produced to allow rapid adaptations. When the target temperature was reached, a constant current is produced to maintain the optimal temperature. **Fig. S26** showed the time-synchronized current and temperature plot during different responsive phases after a sudden change in temperature. During the underheating phase, a rapid increase in output current was generated by the controlled current source in response to the sudden change in temperature. When the target temperature was first reached, the current output remains constant until the actuator was overheated, during which the current output drops again to reach the static phase at which the current levels off.

Supplementary Note S4: Wireless sensing and actuation of a soft sensory robot

Fabrication of Soft Printed *Circuit Board:* A commercially available Pyralux soft PCB material made of polyimide sandwiched between copper was sprayed on and coated with masking paint (Krylon). The paint mask was then partially removed using an infrared laser cutter to expose unwanted copper. Exposed copper was removed by etching in ferrite chloride solution (MG Chemicals 415). The soft PCB was then washed using water and acetone to remove the remaining ferrite chloride and paint mask. Surface mount electrical components including diodes and power regulators were soldered using reflux soldering techniques with solder paste and hot air guns.

Wireless Sensor Data Acquisition: Sensor data readout was carried out using a handheld vector network analyzer (NanoVNA) connected to a commercially available PCB near field probe. Different pressure was applied to the sensor. Port touchstone data was gathered through computer data acquisition software was analyzed using Ansys Electronics. Minimum value of S11 in each trial and the corresponding frequency was acquired using custom Python codes. The frequency response was plotted and was compared to the capacitance value in each trial read directly from the LCR meter.

Wireless Actuation: The inductance of the coil is experimentally measured using a benchtop LCR meter (Matrix MCR-5010). The resonant characteristics of the power transfer network are experimentally measured using a handheld vector network analyzer (NanoVNA). A 50Ω resistive load is connected to the receiving network to simulate the loading of the heater-actuator. The transmission network is connected to a radio frequency power source with a 50Ω characteristic source impedance that consists of a signal generator (Siglent SDG 2042X) connected to an RF power amplifier (EIN 503L). Two high-impedance oscilloscope probes are connected to the transmission network and the resistive load respectively to measure the time-domain waveform using a digital oscilloscope (Siglent SDS 1104X-E). The power delivered by the transmission network and the power harvested at the resistive load, as well as the efficiency of power transfer in the network, were extracted from the time-domain waveform using the following equations:[79-81]

$$P_{Tx} = I_{RMS_{Tx}} V_{RMS_{Tx}} = \left(\frac{\frac{\sqrt{2}}{4} AV_{PP_{Drive}} - V_{RMS_{Tx}}}{R} \right) V_{RMS_{Tx}} \quad (1)$$

$$P_{Rx} = \frac{V_{RMS_{Rx}}^2}{R} \quad (2)$$

$$\eta = \frac{P_{Rx}}{P_{Tx}} \quad (3)$$

Where A is the Amplifier Gain which is equal to 100 for 40dB power amplifier, P_{Tx} is the power delivered by the transmission coil, $I_{RMS_{Tx}}$is the root-mean-squared (RMS) average current through the transmission coil, $V_{RMS_{Tx}}$ is the RMS voltage across the transmission coil, $V_{PP_{Drive}}$ is the peak-to-peak voltage that was fed into the amplifier by the signal generator, $V_{RMS_{Rx}}$ is the RMS voltage across the receiving coil, P_{Rx} is the power received by the receiving coil, η is the efficiency of the power transmission.

The shape deformation including twisting, distorting, and bending are altered and the power transmitted are measured for each situation.

Supplementary Note S5: The effect of size and orientation of the coil on the wireless power transfer

Magnetostatic Finite Element Analysis of Wireless Power Transfer: 3D finite element analysis was performed using commercial software Ansys Maxwell to predict the magnetic field distribution, self-inductance, and mutual inductance of the wireless power transmission networks of different dimensions, horizontal offsets, and distances. The default conductivity, permittivity, and susceptibility of the copper and air derived directly from the materials library of the software were used in the simulations.

Through finite element analysis (FEA) using Ansys Maxwell, Numerical Analysis using MATLAB, and benchtop experimental characterization, we have successfully showcased the influence of various parameters on wireless power transfer (WPT) between receiver and transmitter coils, such as coil distances, horizontal offsets, orientation angles and the size.

Theoretically, the power transmission circuitry can be modeled as shown in **Fig S30**. Here, the internal resistance of the power amplifier, demoted as R, can be considered as the entire internal resistance within the transmission section. Also, the load resistance, R_L, can be considered as the overall internal resistance in the receiving section. Assuming that the transmission and receiving coils share the same inductances (L) and capacitances (C), and resistance R is equivalent to R_L for impedance matching, the total impedance, Z_{eq}, of the power transmission circuitry can be expressed as,

$$Z_{eq} = \frac{\left(j\omega L + \frac{1}{j\omega C} + R\right)^2 + k^2\omega^2 L^2}{\left(j\omega L + \frac{1}{j\omega C} + R\right)} + R \tag{1}$$

in which ω is the angular frequency of the sinusoidal voltage, k is the mutual inductance, L is the inductance of the coils, C is the capacitance of the capacitor in series, and R corresponds to the internal resistance of the power amplifier that is equal to load resistance.

The overall transmitted power delivered to the load resistor at the resonance frequency can be calculated based on the following equation:

$$P_L = V_{S_{RMS}}^2 \left(\frac{1}{\frac{1}{kQ} + kQ}\right)^2 \cdot \frac{1}{R} \tag{2}$$

in which V_{SRMS} is the input voltage, and Q represents the quality factor of the LCR circuitry. The quality factor, Q, can be defined as:

$$Q = \frac{1}{R}\sqrt{\frac{L}{C}} \tag{3}$$

According to the eq (2), the power transmitted at the resonance frequency mainly depends on the coupling factor k and the quality factor Q. Based on eq (3), assuming that L, C, and R remain constant, the quality factor Q remains unchanged. In this case, the power transmission is solely dependent on the coupling factor k (eq. (3)). **Fig. S30B** and **Fig. S30C** present numerical simulation results of the power transmission efficiency as a

function of frequency at various coupling factors ranging from 0.1 to 0.8. It is observed that the power transmission efficiency is suppressed at the resonance frequency at very high cooping factors (from 0.6 to 0.8). The maximum power transfer occurs at two other frequencies, one higher and oner lower than the resonance frequency. This phenomenon, known as “frequency split”, has been reported in previous studies as well [92,93]. Conversely, for relatively lower coupling factors (from 0.1 to 0.4), the transmission efficiency reaches its maximum at the resonance frequency and decreases as the coupling factor decreases.

Furthermore, We conducted computational and experimental investagtaions to analyze the influence of separation distances between reciving and emission coils, horizontal offsets, orientation angles on the power transfer efficiency (**Fig. S31**). As expected, an increase in separation distance, horizontal offeset and orentation angle leads to a decrease in the power transfer efficiency. The impact of coil size on the power transmission is relatively more complex, compared to the previous parameters. The transmitting and receiving coil’s surface area plays a crucial role in enclosing the magnetic field lines, consequently affecting the magnetic flux. Hence, both the self-inductance and the mutual inductance of the coil increase as the area of the coil increases, which has been validated through FEA, as shown in **Fig S32B** and **Fig. S32C**. We adjusted the series capacitor used in the frequency-tuning circuit for different-sized coils to achieve a consistent resonance frequency. The experimental measurement of power transmission is shown in **Fig. S32D**. It is observed that, the size significantly affects the power transfer efficiency.

Supplementary Note S6: Application-oriented device customization

Figs. S38&**S39** present the adaptability, geometrical adjustments, and the integration of various functional units, enabling the seamless integration with the organic tissues it is intended to interact with. For example, **Fig. S38** shows the design parameters of the starfish-like implant, highlighting the flexibility in adjusting the number of legs, as well as their respective width and lengths, to comply with the specific demands of varied applications. **Fig. S39** presents a diverse range of devices, exhibiting versatility in both shape and dimensions. These devices designed for their specific applications, can be safely delivered using either catheters or needles, highlighting the potential for minimally invasive implantation procedures. Notably, owing to its flexibility, the wireless antenna can be conveniently rolled to fit within the catheter, demonstrating adaptability and integration without comprising the function of either component.

Medical catheters are available in various sizes to accommodate different medical requirements, especially for minimally invasive procedures. For instance, Woodington et al designed a soft electronic device capable of being rolled to fit into a 14-gauge Tuohy needle (Diameter ~1.6mm) and navigated up the spinal column (aspect ratio ~378) [105]. Similarly, Whyte et al reported a therapeutic epicardial device navigable through a 14-gauge needle (aspect ratio ~8) [106]. In addition, Chen et al reported a magnetoelectric implant can be delivered percutaneously via a catheter for endovascular stimulation of peripheral nerves (aspect ratio ~2.6) [107]. Montgomery et al developed a cardiac patch (width ~1cm) capable of being delivered through an orifice as small as 1mm (aspect ratio ~125) [110]. Further innovations include a flexible neural device, which can be wrapped around a needle tip (Diameter ~0.8mm) and inserted into the primary motor cortex (aspect ratio ~1.8) [109]. Liu et al pioneered a method for syringe-injectable electronics, capable of being folded and crumpled to fit inside a syringe, facilitating insertion into the deep brain (aspect ratio ~500) [108]. Additionally, Xie et al demonstrated a 3D-macroporous device capable of spontaneously curving into a cylindrical surface and being syringe-injected through needles into brain tissues (aspect ratio ~31.4) [111]. Lastly, there is an integration of an electrocorticography system featuring a large area, assisted with a soft robotic actuator, can be surgically implanted on the cortex through a small burr hole (aspect ratio ~4) [104]. **Table S2** compares the structural adaptability and customizability of our design with those reported in recent studies, to further showcase our implantable robots can be highly adaptable and tailored for minimally invasive insertions.

Supplementary Note S7: *In vivo* animal test of a soft robotic thera-gripper

All procedures follow the standard on Rodent Survival Surgery issued by the UNC IACUC (ID 21-241.0). In brief, instruments are autoclaved for sterile surgery, with a bead sterilizer used between subsequent animals. The surgeon will scrub hands with disinfectant soap and use a mask as a barrier between the surgeon's face and the microscopic field. Upon aesthetic induction, toe pinch is used to determine lack of response to an external stimulus. Lubricant eye ointment is plied to prevent drying the eyes. Mouse body temperature is monitored with a rectal probe and maintained at 37 ^{o}C throughout the surgical procedure. All operative procedures are done aseptically with sterilized instrumentation and materials: hair is removed from the incision area by application of an alkali cream (Nair), the area is cleansed with a povidone iodine solution, then swabbed with 70% alcohol. Most surgeries are done with operating microscopic view. Post-operatively the animal is kept on a water-jacketed warming pad and monitored constantly until they regain sternal recumbence, after which they are housed in a recovery cage and observed frequently (3-4 times per hour) until they have recovered to normal activity.

Myocardial Infarction (Left coronary artery ligation): Under anesthesia, an endotracheal tube is placed through the mouth and into the larynx; and the animal is mechanically ventilated (for a 25 g mouse, 150 breaths/minute and 250 uL/breath). A thoracic skin incision paramedial to the sternum is made; and the 3rd and 4th ribs are separated using the retractor to expose the ligation site of the left coronary artery. A 7-0 suture is placed in the myocardial wall around the artery and tied permanently to induce myocardial infarction. On day 14 post-surgery, heart contraction and relaxation is monitored using a soft robotic thera-gripper and echocardiography. Based on the echocardiography, we collect B-mode and M-mode images. The M-mode images are obtained from short axis B-mode images by placing the M-mode sample gate perpendicular to the LV walls. We can calculate the fractional shortening (FS) that is one of the most commonly used parameters to assess the LV systolic function based on M-mode tracings (**Fig. S50B**) [124,126]:

$$FS(\%) = \frac{LVEDD - LVESD}{LVEDD} \times 100$$

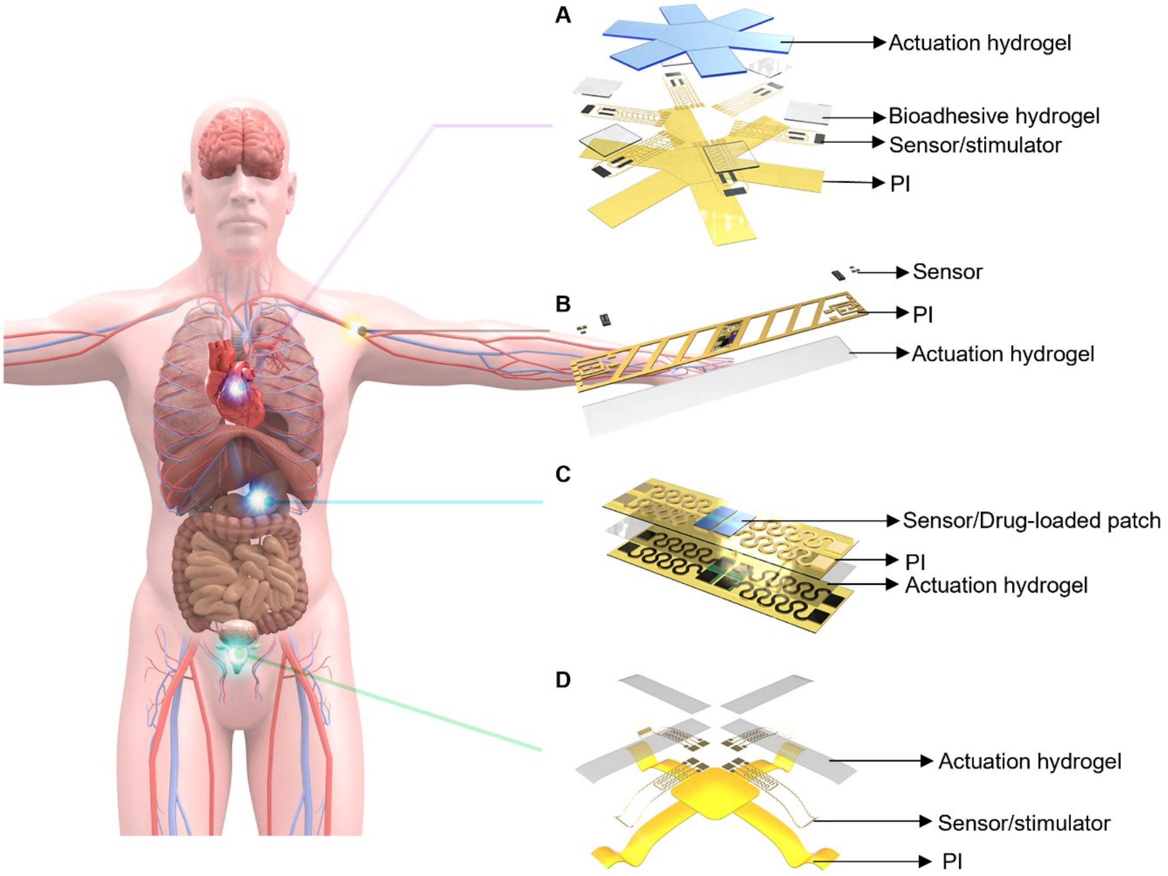

Fig. S1. Schematic illustration with exploded views of the soft sensory robots for diagnosis, stimulation, and drug delivery. (**A**) A soft robotic epicardial gripper grasping a beating heart to quantify cardiac contractility and temperature and apply coordinated electrical stimulation. (**B**) A soft robotic cuff twisting around the vessel for monitoring blood pressure and proving structural support. (**C**) An ingestible robot expanding in the stomach for monitoring gastric pH and delivering drugs over long periods. (**D**) A soft robotic bladder gripper wrapping around a bladder for monitoring its volume change in real-time and providing timely stimulation to sacral nerve. Here, all the robotic systems seamlessly integrate two primary components together: (1) an actuator made of a passive polymer layer (e.g., polyimide, PI) and an active hydrogel layer offers stimuli-responsive actuation in biological environment, and (2) embedded biosensors that provide in situ and real-time health monitoring.

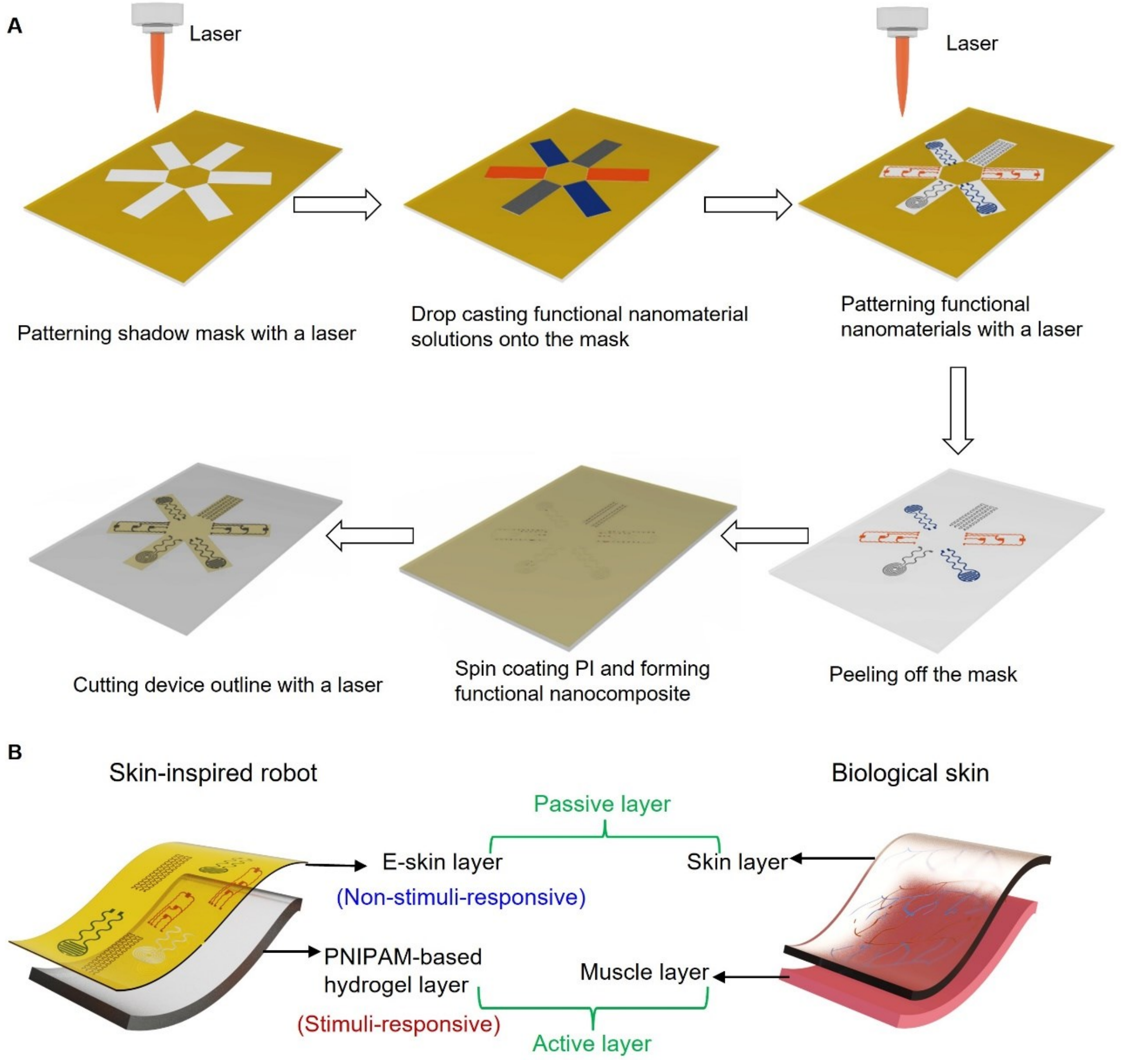

Fig. S2. A soft starfish-like robot based on an active hydrogel layer and a passive nanocomposite layer. (**A**) Schematic illustration showing fabrication process of the flexible multifunctional nanocomposite film that fully integrates biosensors into the robotic structure. (**B**) Schematic illustration showing the bilayer design of the sensory robot: a non-stimuli-responsive layer made of functional nanocomposites, and a thermal-responsive active layer made of the PNIPAM-based hydrogel, mimicking the biological skin and muscle, respectively.

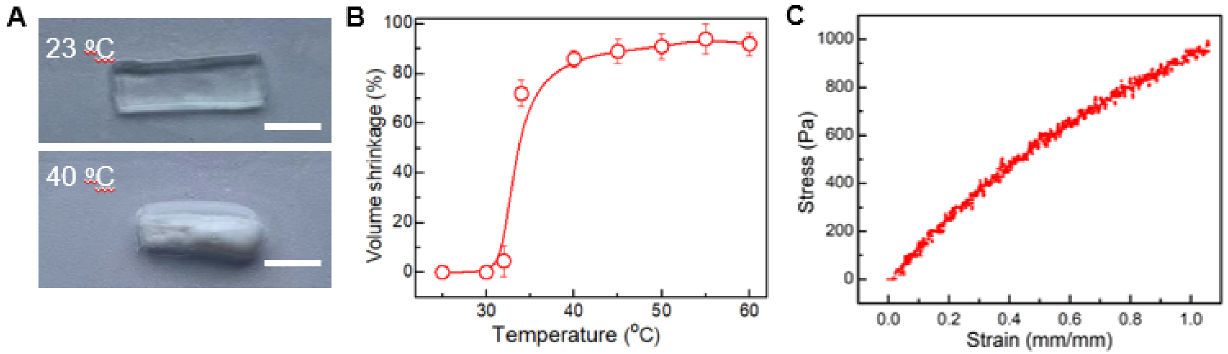

Fig. S3. Characterization of thermo-responsive hydrogel (e.g., poly(N-isopropylacrylamide), PNIPAM). (**A**) Optical images of a PNIPAM hydrogel undergoing coil-globule transition as the temperature changes from 23 °C to 40 °C. Scale bars, 7 mm. (**B**) Measured volume shrinkage of PNIPAM hydrogel during a cooling process with temperature changing from 60 °C to 25 °C. (**C**) Tensile stress curve of PNIPAM hydrogel (Dimensions: thickness 4mm, width 10mm, initial length 15mm), highlighting an elastic modulus (E) of approximately 1.2 kPa. This value is within the range of soft tissues, which typically exhibit an E from a few kPa to hundreds of kPa, ensuring the hydrogel's compatibility with biological tissues and reducing risks associated with mechanical mismatches.

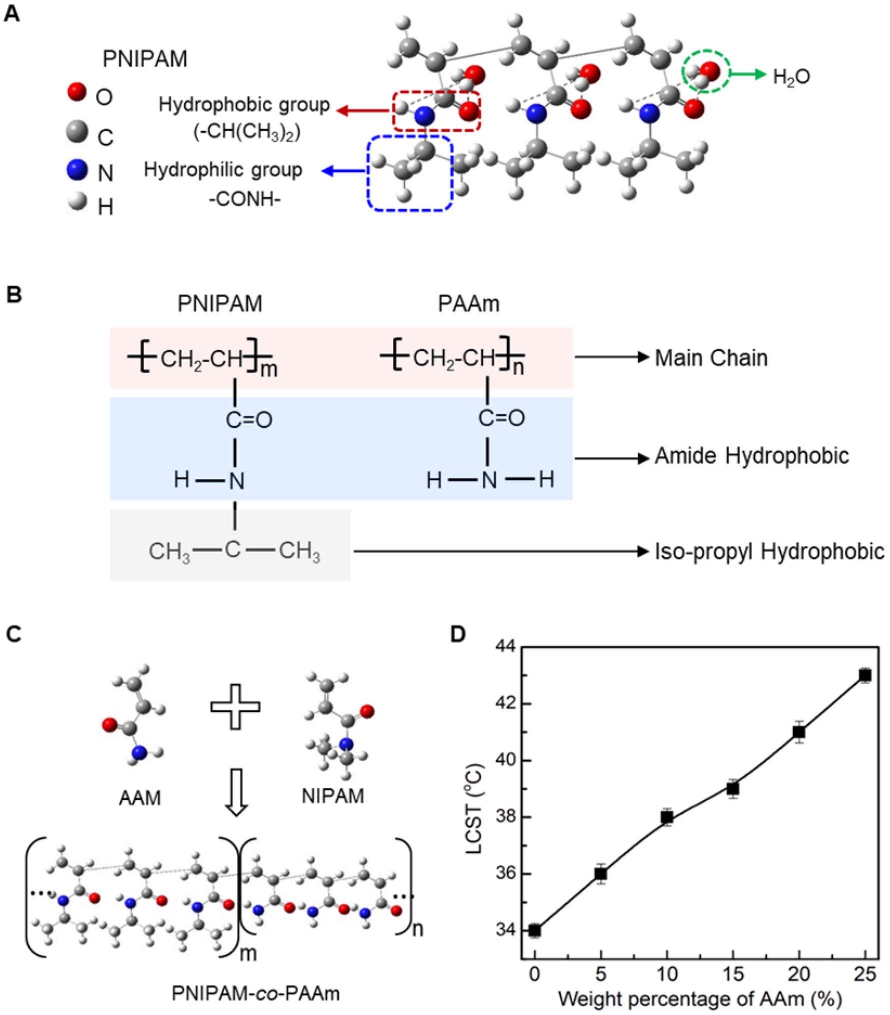

Fig. S4. LCST tunability of PNIPAM-based hydrogel. (**A**) A schematic representation of PNIPAM featuring hydrophobic isopropyl (-CH(CH_3)$_2$) side groups and hydrophilic amide (-CONH-). (**B**) A schematic representation of a NIPAM monomer and an AAm monomer. (**C**) A schematic representation of the synthesis of a P(NIPAM-AAm). (**D**) Effect of AAm concentration on LCST behavior of PNIPAM-*co*-PAAm.

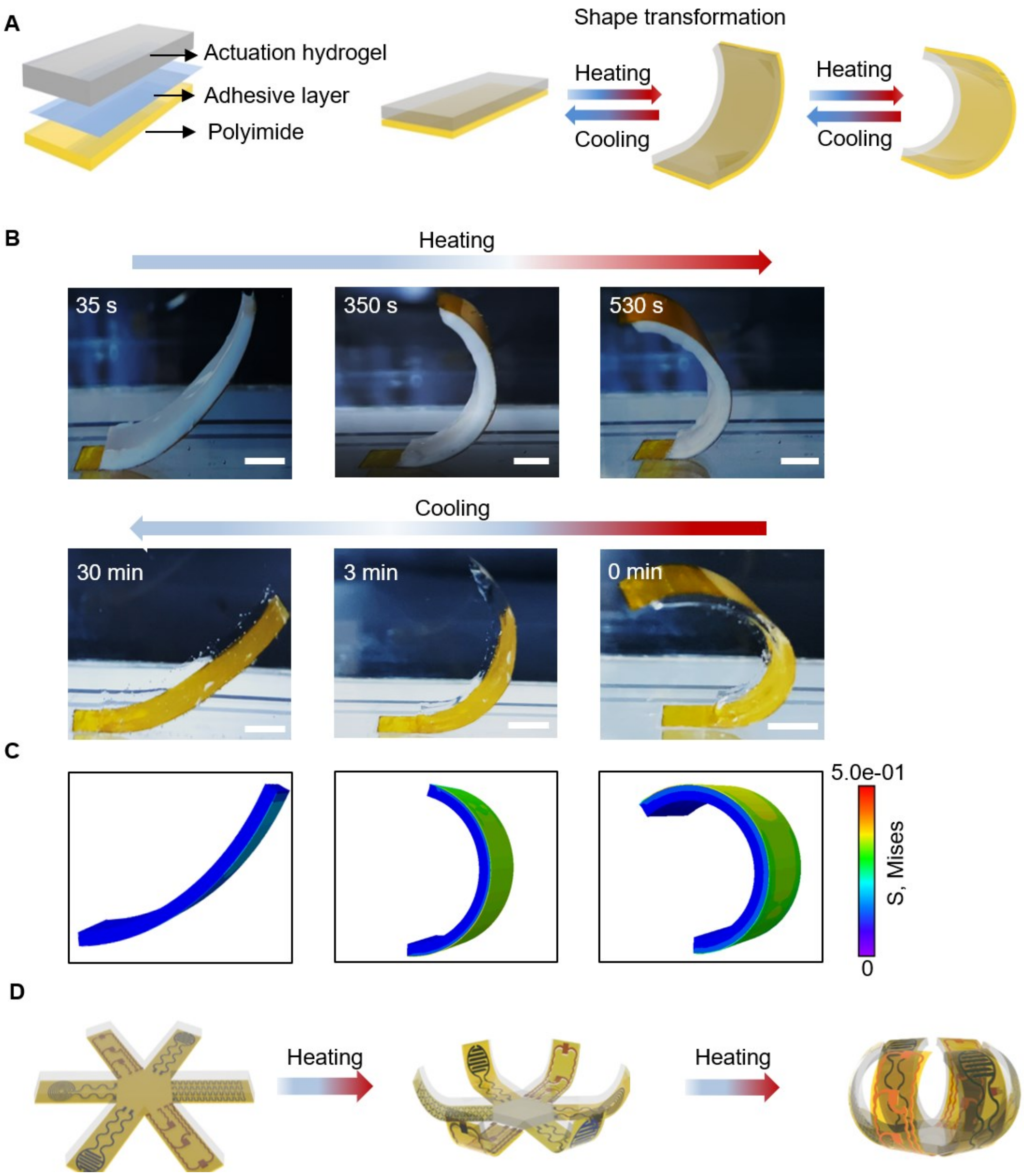

Fig. S5. Design of a bilayer actuator made of a passive biocompatible-polymer layer (e.g., polyimide) and an active thermo-responsive hydrogel layer (e.g., poly(N-isopropylacrylamide), PNIPAM). (**A**) Schematic illustration showing reversible bending motion of a bilayer soft robot during heating and cooling process. (**B**) Optical images showing a bilayer soft robot that bends reversibly as the temperature of the system moves up and down at the lower critical solution temperature (LCST) (~34 °C). scale bars, 5 mm. (**C**) Finite element modeling of structural transformation corresponding to the states shown in (**B**). (**D**) Schematic illustration of the starfish-like soft robot with multifunctional rays actuated by temperature change. Scale bars, 5 mm.

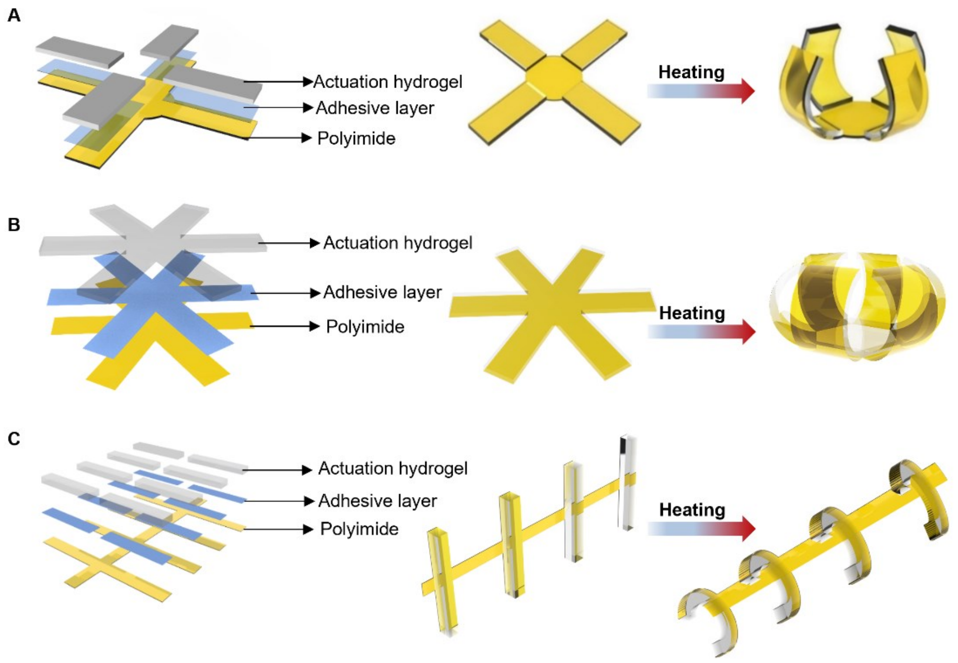

Fig. S6. Schematic illustrations of three representative shape-morphing systems made of PNIPAM/PI bilayers with programmable motions. (**A**) Soft starfish-like structure with four arms. (**B**) Soft starfish-like structure with six arms. (**C**) Soft fishbone-like structures with four pins.

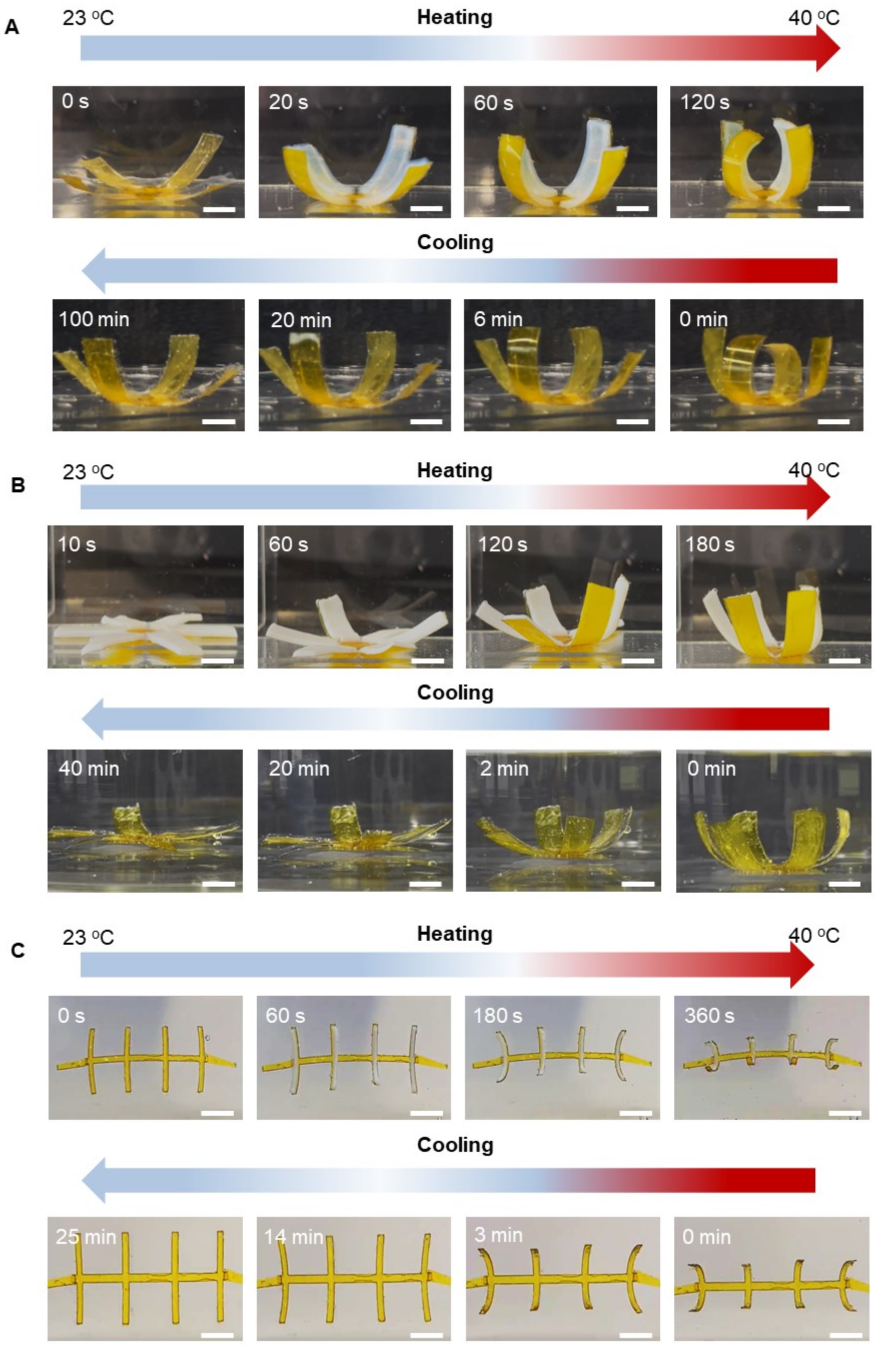

Fig. S7. Reversible shape transformation of three robotic systems based on the design of PNIPAM/PI bilayers. (**A&B**) Soft starfish-like robots with four and six arms, respectively, can extend and bend their arms for locomotion in response to temperature change. (**C**) A soft fishbone-like robot with peripheral pins undergoing open-close motion via periodical thermal stimulation. scale bars, 5 mm.

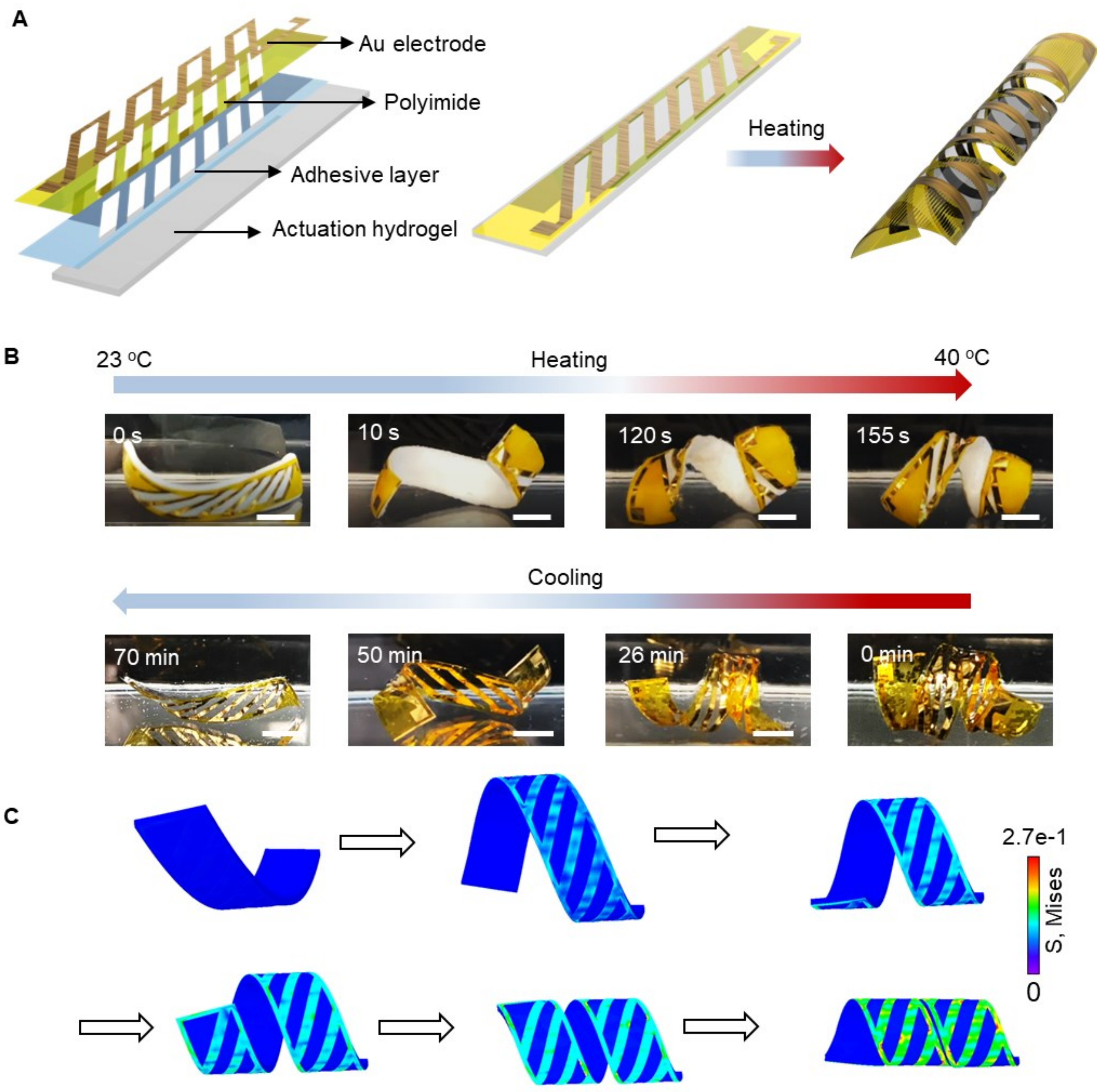

Fig. S8. Soft robot with a helical bilayer of PNIPAM and heater-integrated PI mimicking a chiral seedpod. (**A**) Schematic illustration showing the nature-inspired design of a bilayer ribbon that can undergo a twisting motion. Here, the Au resistor coated on the PI layer can serve as a thermal heater, which represents the capability of functional integration. (**B**) Optical images of the robot undergoing a reversible twisting transformation in response to temperature change. The PNIPAM hydrogel contracts while PI maintains relative stability upon heating. The contraction of the PNIPAM hydrogel activates a tilting torque through the bonded PI strips, resulting in asymmetric deformation throughout the device. Scale bars, 5 mm. (**C**) FEA simulation results of a bilayer soft robotic device mimicking a chiral seedpod. Stress distribution across the device during the shape-shifting process, facilitated by strategic stripe patterns on the 3D bilayer robot. This visualization highlights the material property mismatches including mechanical and thermal responsiveness that drive the device's complex morphological transformations.

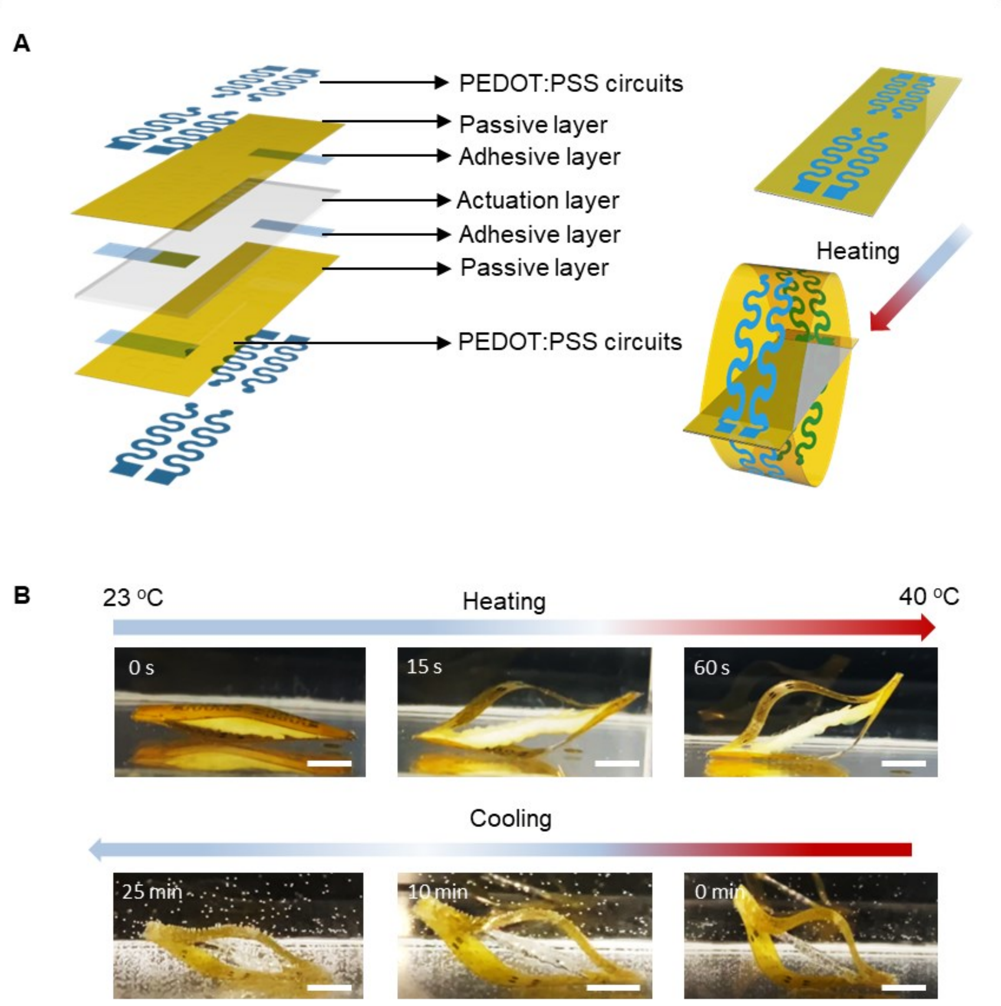

Fig. S9. Soft robot integrated flexible PEDOT:PSS/PI nanocomposite electronics to enable sensing functionality. (**A**) Schematic illustration showing the exploded view and structural transformation of the robot. Here, the PEDOT:PSS/PI nanocomposite film acts as the passive layer, offering both the structural support and the sensing functionality for the robot. (**B**) Optical images of reversible shape transformation of the sensory robot over time by heating and cooling. The temperature increase triggers the PNIPAM hydrogel to contract, hence pushing the PEDOT:PSS/PI nanocomposite layers to arch outwards, and vice versa. Scale bars, 5 mm.

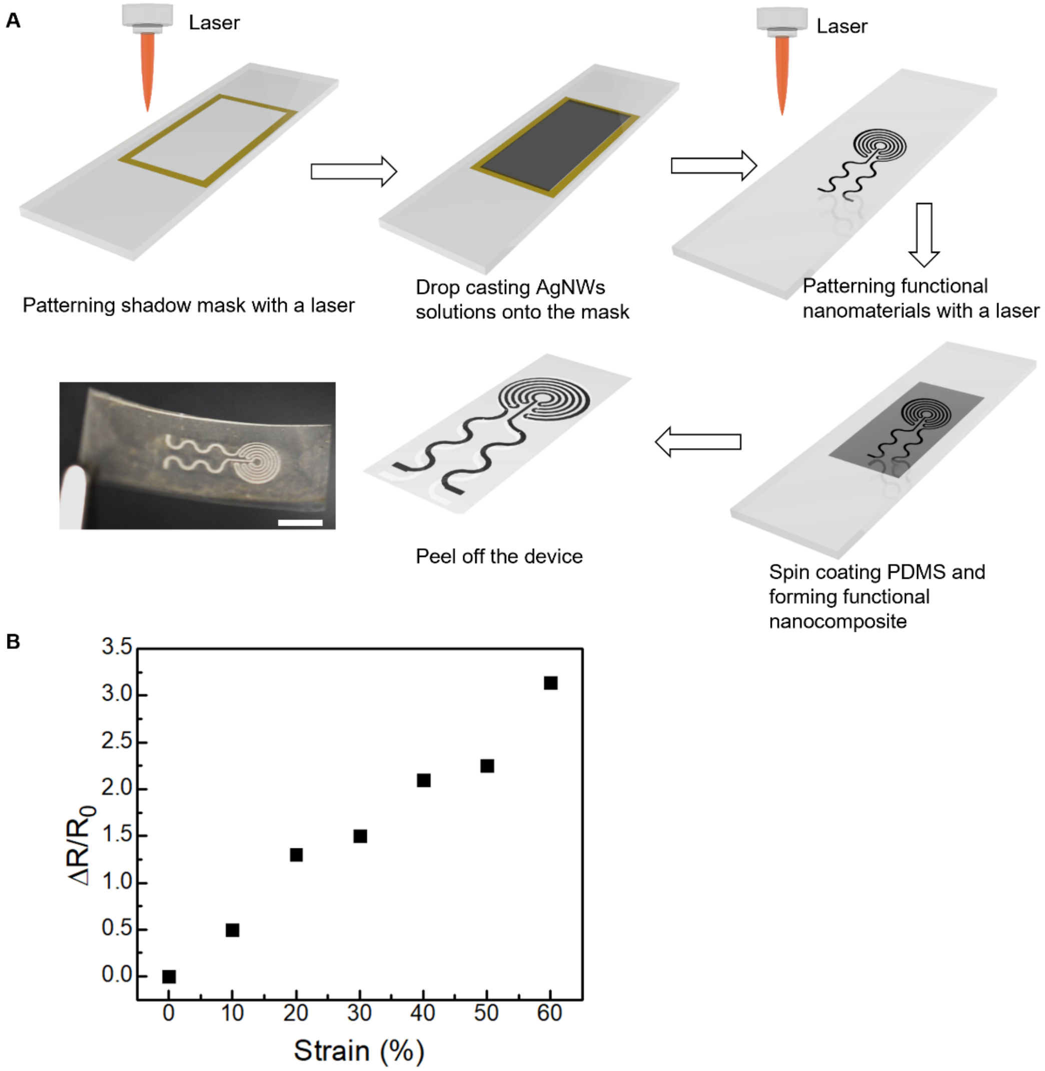

Fig. S10. ***In situ*** **integration of AgNWs/PDMS-based strain sensor.** (**A**) The fabrication process of an AgNW/PDMS-based strain sensor. (**B**) Relative resistance changes of the AgNWs/PDMS strain sensor toward the applied strain. Scale bar, 2 mm.

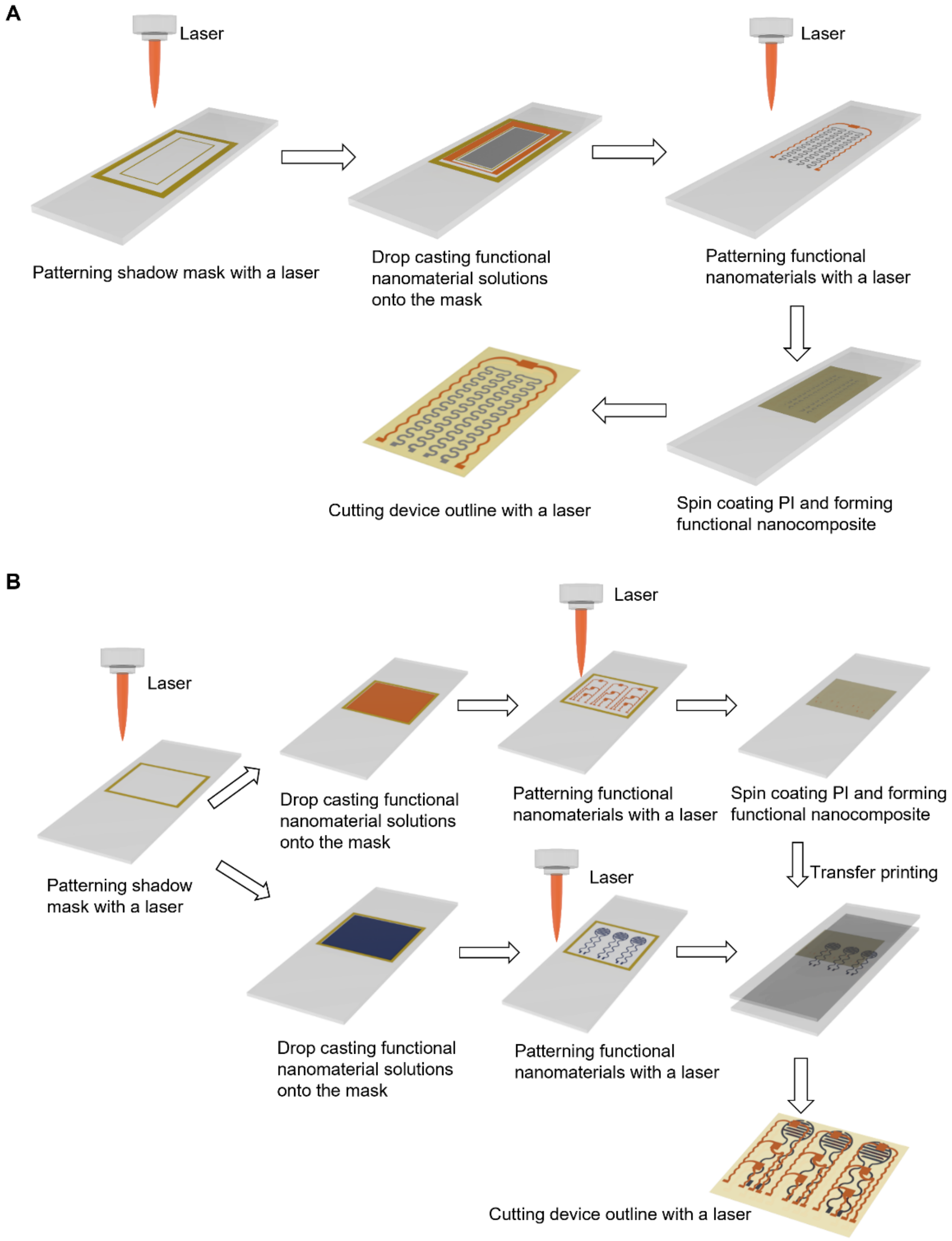

Fig. S11. Schematic illustration for anisotropic integration of multiple nanocomposite materials to form a multi-modal sensing system. (**A**) The fabrication process of a multi-modal sensing system containing an RGO/PI-based temperature sensor and an AgNW/PI-based heating pad. (**B**) The fabrication process of a multi-modal sensing system containing a PEDOT:PSS/PI electrodes and RGO/PI temperature sensors.

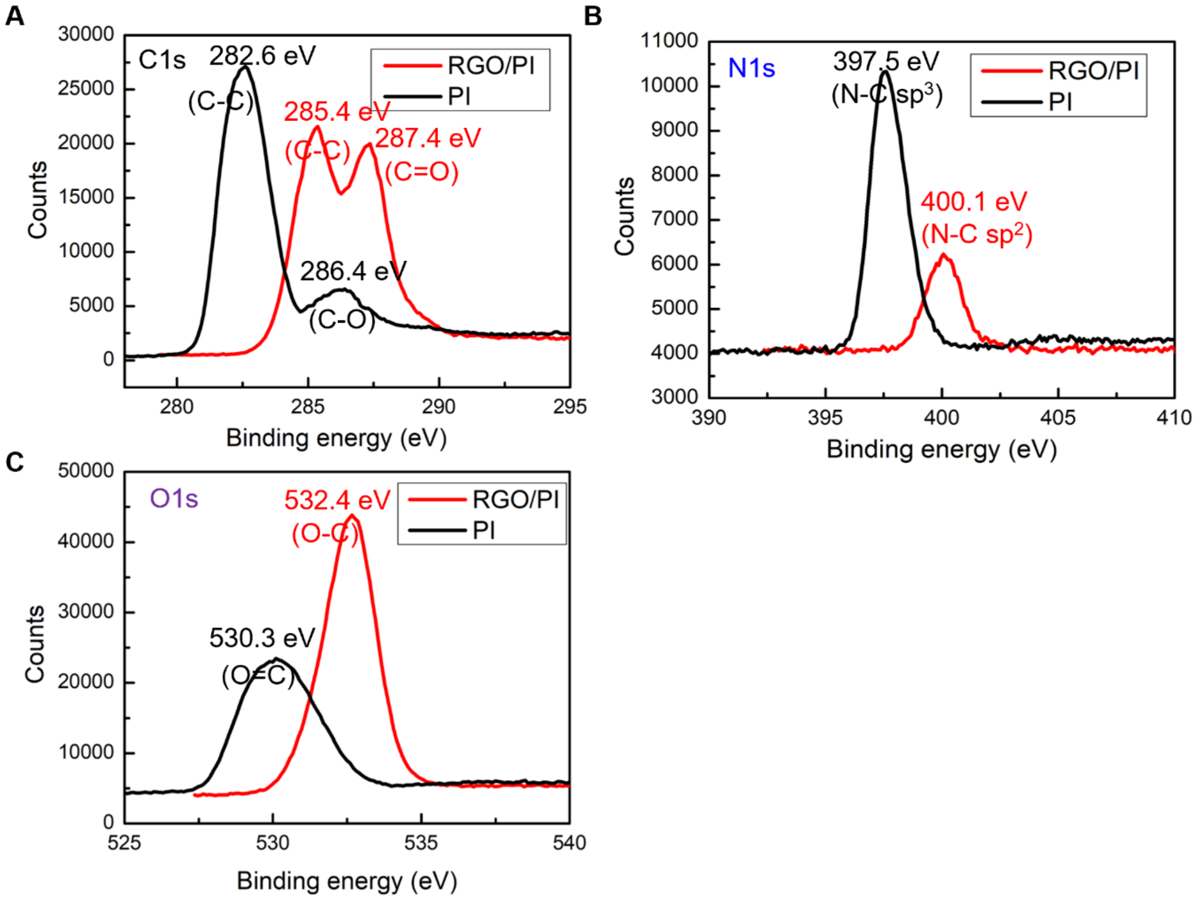

Fig. S12. Analysis of X-ray photoelectron spectroscopy (XPS) of RGO/PI-based nanocomposite films. (**A**) C1s, (**B**) N1s and (**C**) O1s spectra of pristine PI film and RGO/PI nanocomposite film.

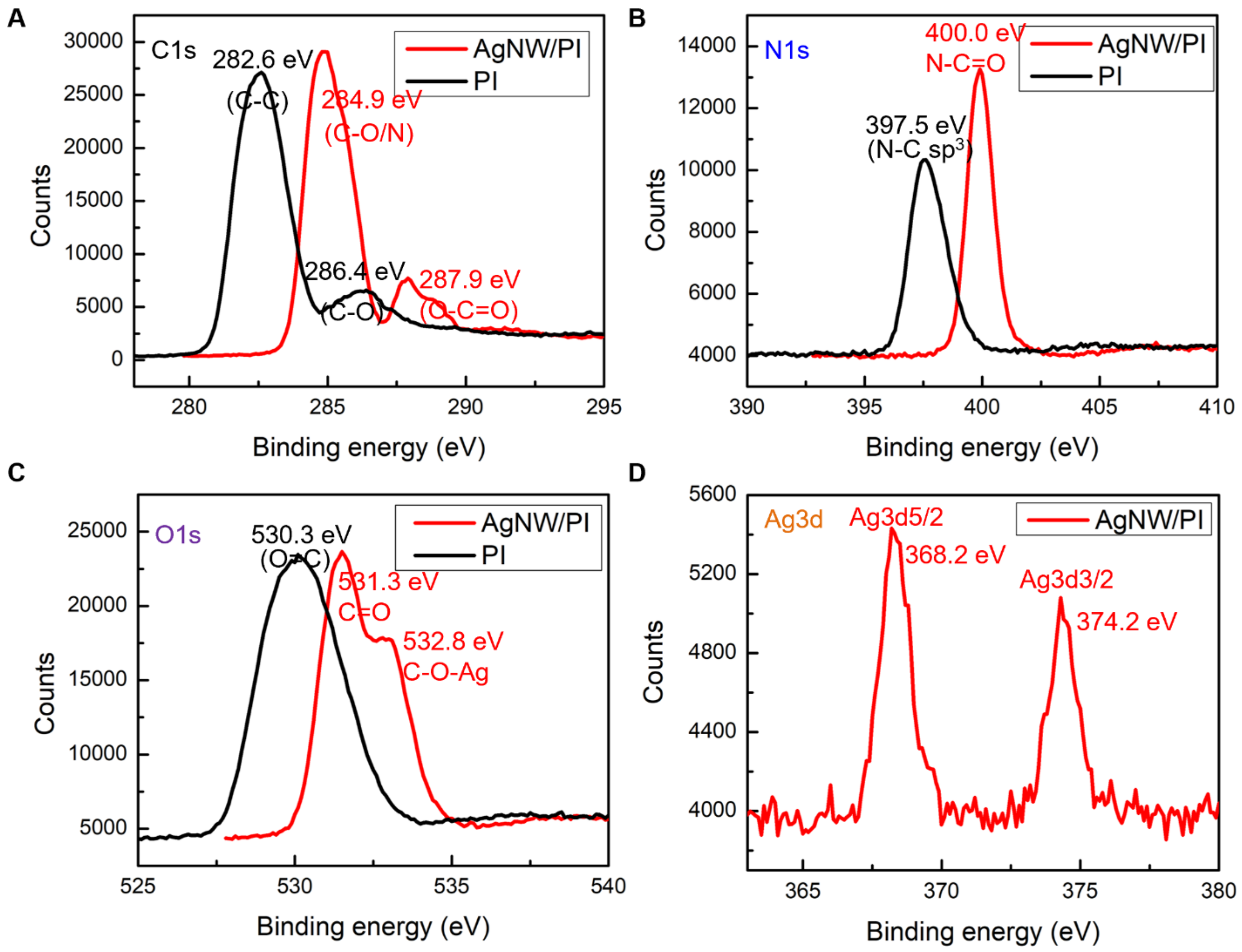

Fig. S13. Analysis of X-ray photoelectron spectroscopy (XPS) of AgNW/PI-based nanocomposite films. (**A**) C1s, (**B**) N1s, (**C**) O1s and (**D**) Ag3d spectra of pristine PI film and AgNW/PI-based nanocomposite film.

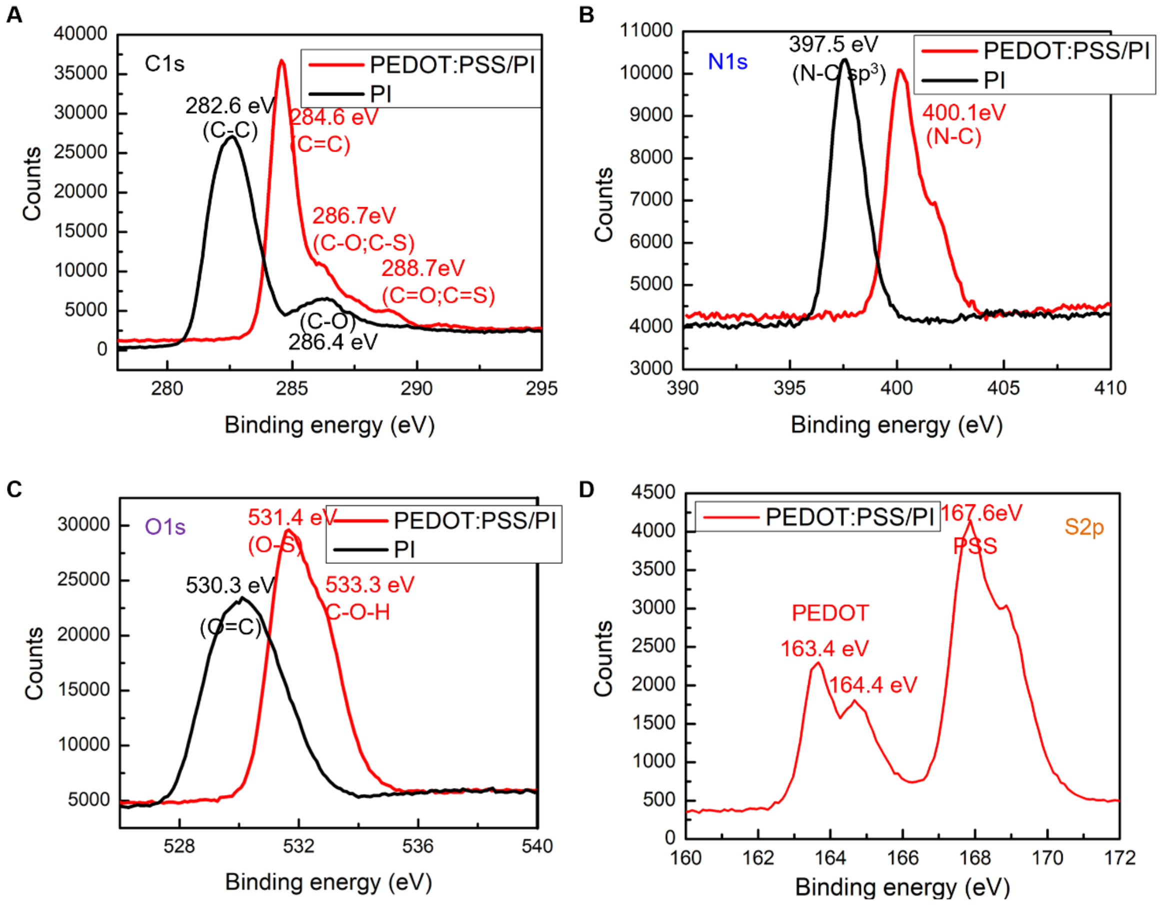

Fig. S14. Analysis of X-ray photoelectron spectroscopy (XPS) of PEDOT:PSS/PI-based nanocomposite films. (**A**) C1s, (**B**) N1s, (**C**) O1s, and (**D**) S2p spectra of pristine PI film and PEDOT:PSS/PI nanocomposite film.

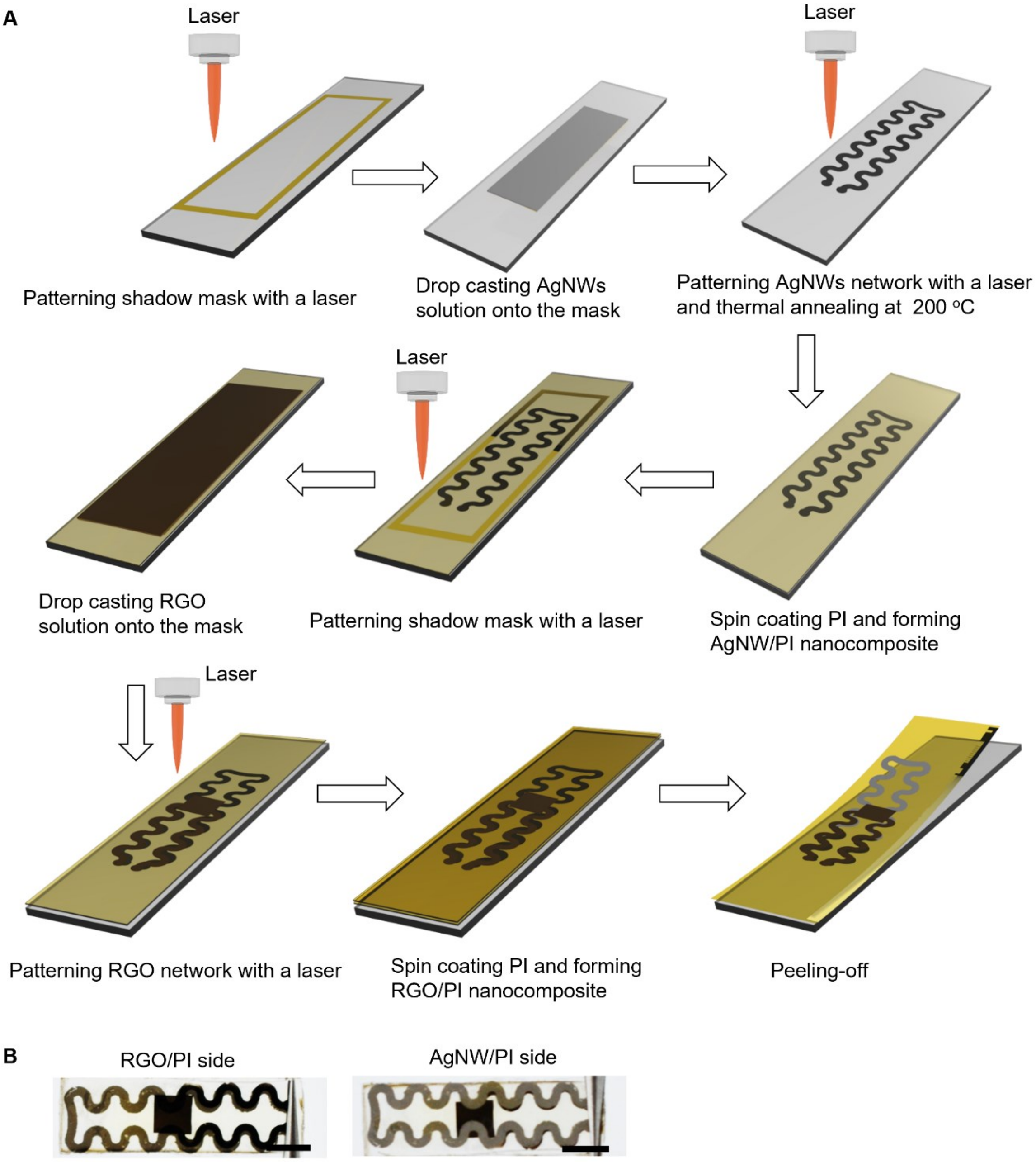

Fig. S15. Anisotropic integration to enable layer-by-layer stacking of functional nanocomposite. (**A**) Schematic illustration shows the fabrication process of layer-by-layer stacking of structured functional nanocomposite. Here, a thin PI film (thickness ~10 μm) firmly stacks an AgNW/PI-based heater on top and an RGO/PI-based thermal sensor on bottom. (**B**) The optical image of the integrated system that firmly stacks the electrical heater and the temperature sensor with a thin PI film. Scale bars, 5 mm.

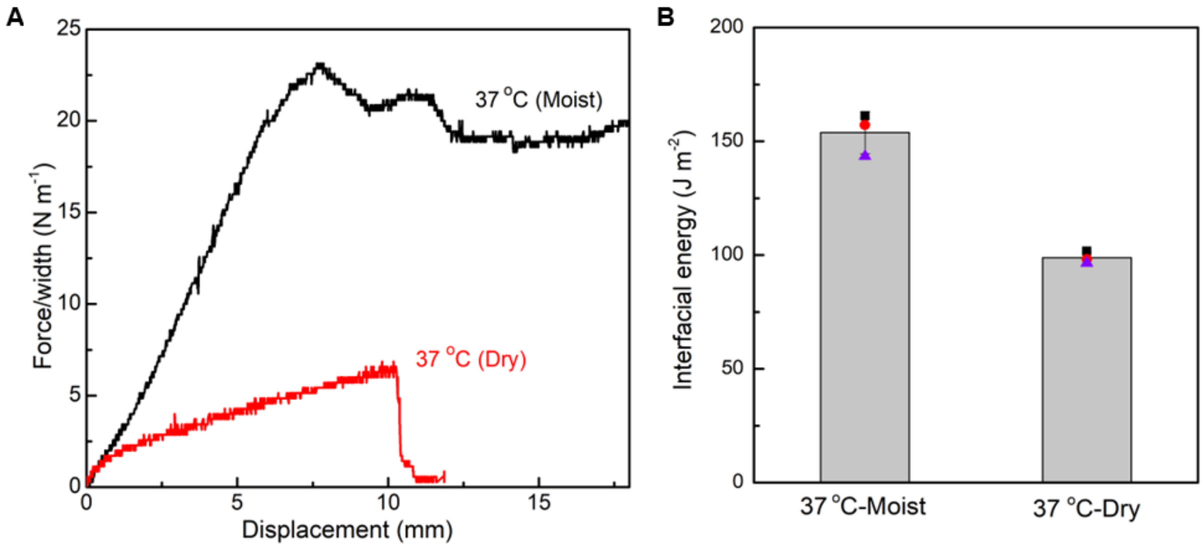

Fig. S16. Interfacial adhesion characterization via peeling tests. (**A**) Adhesive force-displacement curves and (**B**) calculated interfacial energy from the 180° peeling test of the bio-adhesive layer interacting with hydrogel. The adhesion force is higher under a moist condition, mimicking the human body environment, favorable for potential implantable biomedical device applications.

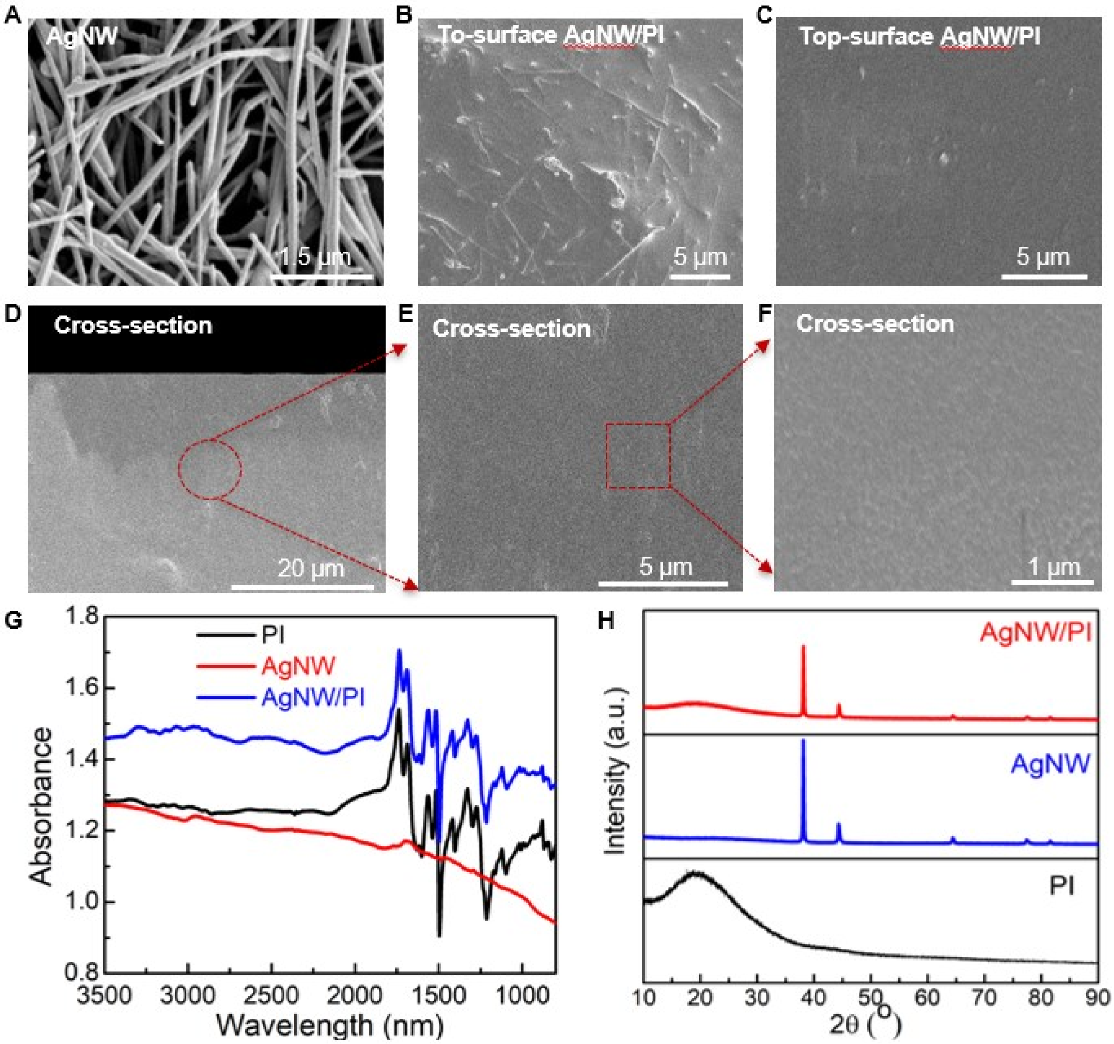

Fig. S17. Characterization of an AgNW/PI-based heater. (**A**) SEM image of as-synthesized AgNWs. (**B**) SEM image of AgNW/PI nanocomposite with partial PI infiltration. (**C**) Top-surface SEM image of AgNW/PI nanocomposite with full PI infiltration. (**D**-**F**) Cross-sectional SEM images of AgNW/PI nanocomposite, revealing the complete penetration of PI throughout the AgNW matrix with the absence of voids. (**G**) Analysis of Fourier-transform infrared spectroscopy (FTIR) on the AgNW/PI nanocomposite. (**H**) Analysis of X-ray powder diffraction (XRD) on the AgNW/PI nanocomposite.

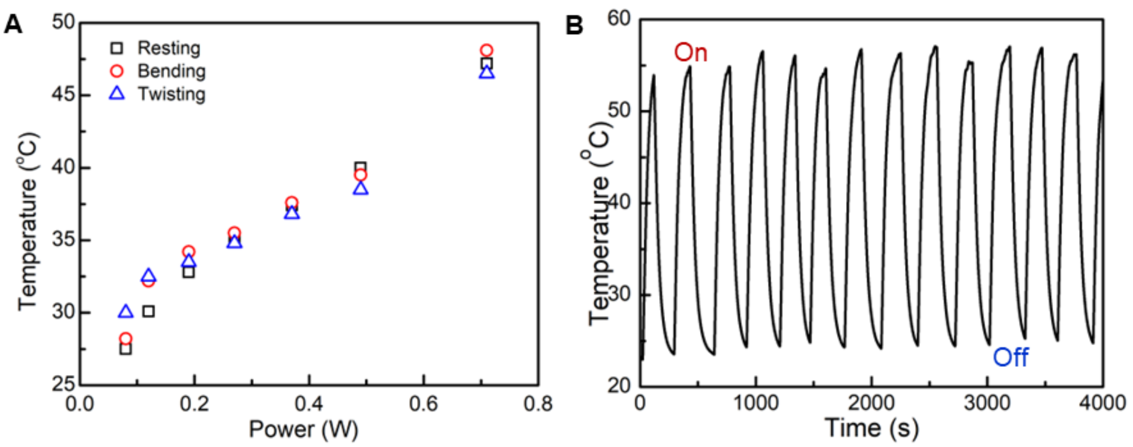

Fig. S18. Electrothermal characterization of an AgNW/PI-based heater. (**A**) Measured surface temperature of the AgNW/PI nanocomposite heater as a function of the input power while the heater is in resting, bending and twisting state. (**B**) Temperature response of the AgNW/PI-based heater over 10 cycles of heating.

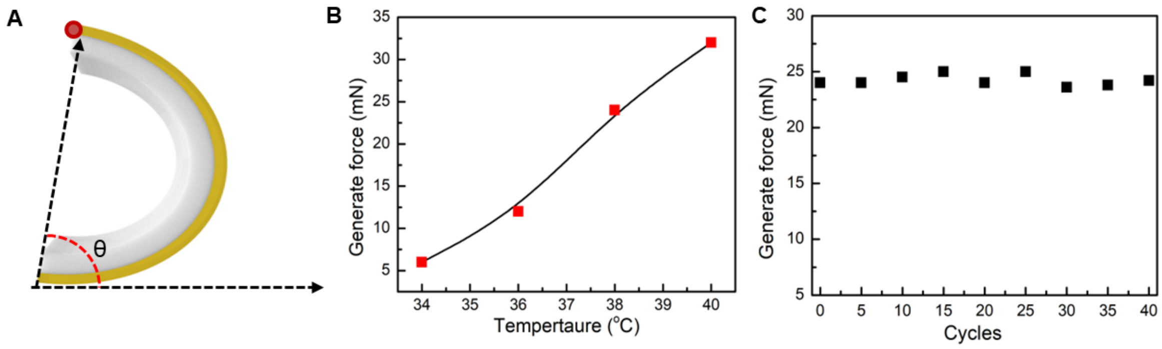

Fig. S19. **Actuation behavior of bilayer soft robots under ambient conditions.** (**A**) The schematic illustration of the bending angle *θ*. (**B**) Measured static force changes of the soft robotic finger under different temperatures induced by different electrical powers. (**C**) Measured static force changes as a function of number cycles. Here, all characterizations were conducted under ambient conditions because of the unique temperature-responsive nature of the PNIPAM hydrogel. Its actuation force and shape morphing capabilities are predominantly influenced by temperature-induced molecular structural changes, rather than slight humidity changes typical in human body environments. This characteristic underlines the hydrogel's efficacy in translating thermal stimuli into mechanical actions, essential for the intended biomedical applications.

Table S1 Parameter comparison of different hydrogel-based actuators.

Hydrogel system	Actuation trigger	Bending speed	Generated force	Ref.
Bulk PNIPAM	Thermal		12.5 mN (42 °C)	[68]
PNIPAM/GO	Thermal	10°/s (50 °C)		[67]
GO/Poly(N,N-dimethylaminoethyl methacrylate)	Thermal	86°/min (60 °C)	0.9 mN (65 °C)	[69]
PNIPAM-Laponite/PNAGA	Thermal	3.8°/s (45 °C)		[74]
PNIPAM/K6APA	Thermal	1.4°/s (40 °C)		[70]
PNIPAM-PDMAPMA	Thermal	4.5°/s (60 °C)		[71]
P(AAc-co-AAm)/P(AAc-co-NIPAM)	Salt	7°/s (4M saline)	150 mN (4M saline)	[72]
MXene-cellulose	NIR-light	26°/s (80mW/cm^2)	43.4 mN (50 mW/cm^2)	[73]
PNIPAM/Multi-functional nanocomposite	Thermal	5°/min (40 °C)	32 mN (40 °C)	This work

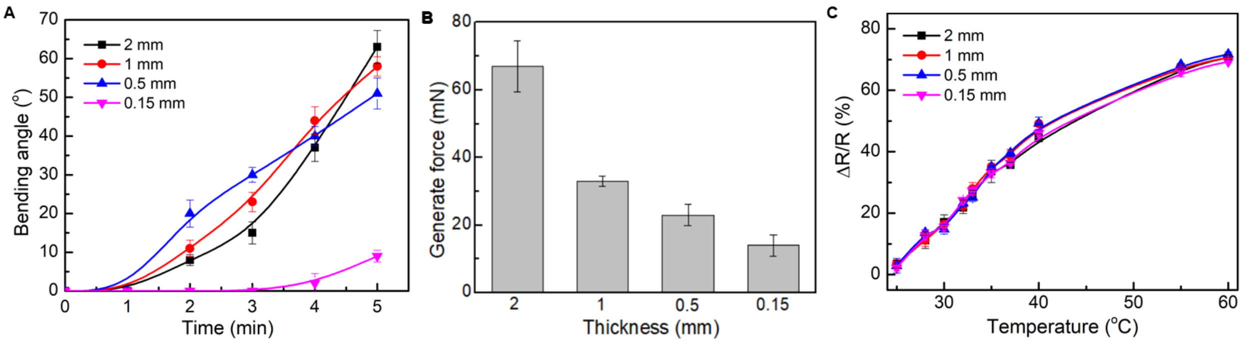

Fig. S20. Effect of hydrogel thickness on actuation performance. (**A**) Bending angle over time for varying hydrogel thicknesses at 41 ºC. (**B**) Measured static force changes at 41 ºC across different hydrogel thicknesses. (**C**) Resistive response of a thermal sensor (ERT-J0ET102H) across a temperature range of 22 ºC to 60 ºC for different hydrogel thicknesses.

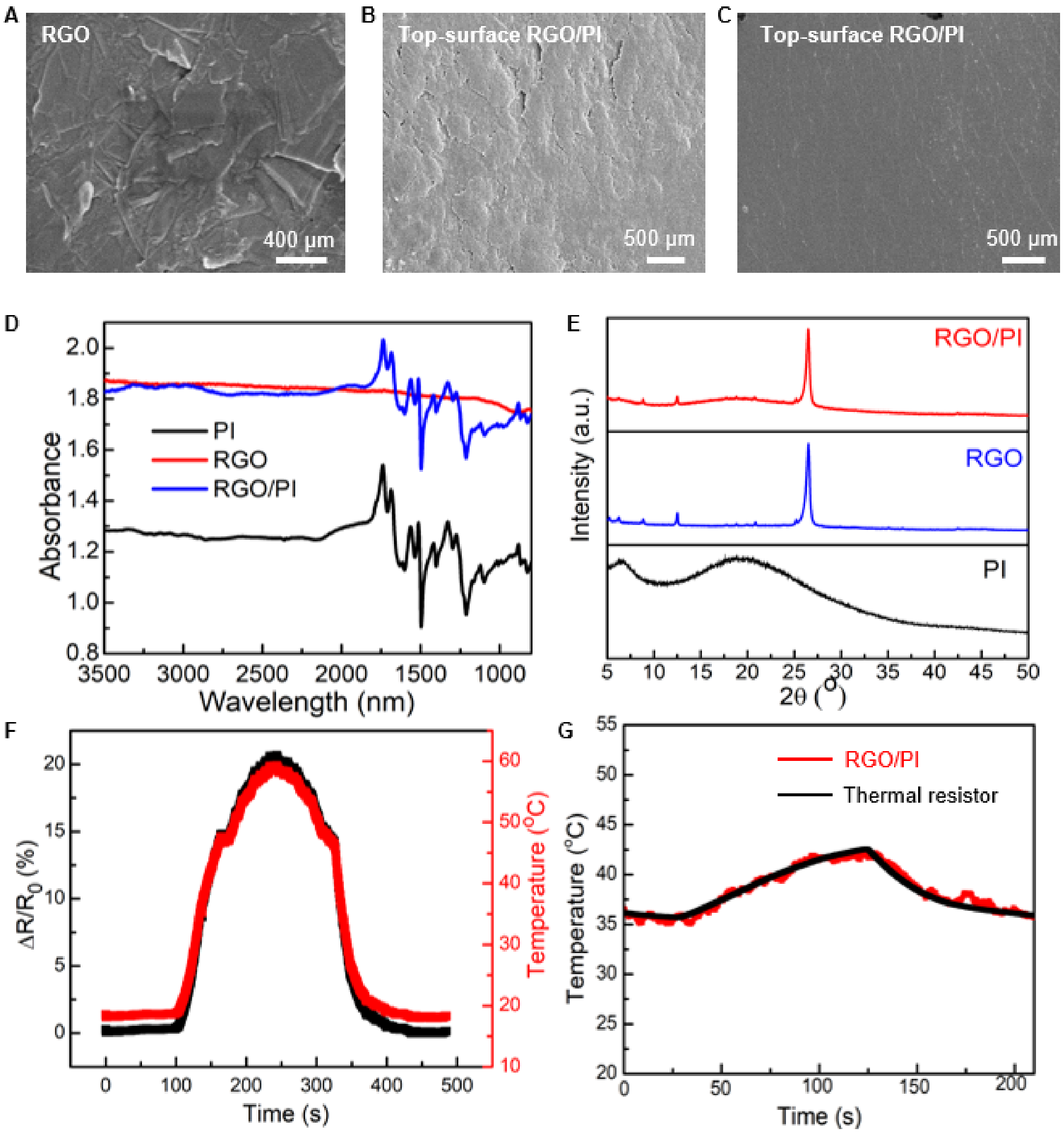

Fig. S21. Characterization of RGO/PI-based temperature sensor. (**A**) SEM image of pristine RGO. (**B**) SEM image of RGO/PI nanocomposite with partial PI infiltration. (**C**) SEM image of RGO/PI nanocomposite with full PI infiltration. (**D**) Analysis of Fourier-transform infrared spectroscopy (FTIR) on the RGO/PI nanocomposite. (**E**) Analysis of X-ray powder diffraction (XRD) on the RGO/PI nanocomposite. (**F**) Results of temperature measurement using RGO/PI-based temperature sensor. (**G**) Temperature measurement on the RGO/PI thermal sensor and the commercial thermal resistor.

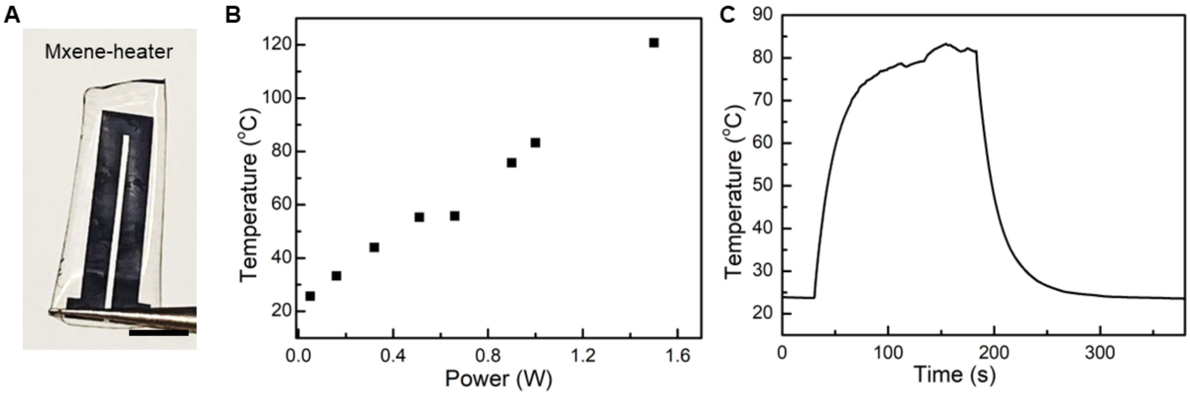

Fig. S22. MXene-based nanocomposite heater for the e-skin layer of soft robot. (**A**) Optical image of a flexible MXene/PI nanocomposite electrothermal heater. (**B**) Measured surface temperature of the MXene/PI nanocomposite heater as a function of the input power. (**C**) Temperature response of MXene/PI heater under an input power of 1.1 W. Scale bar, 5 mm.

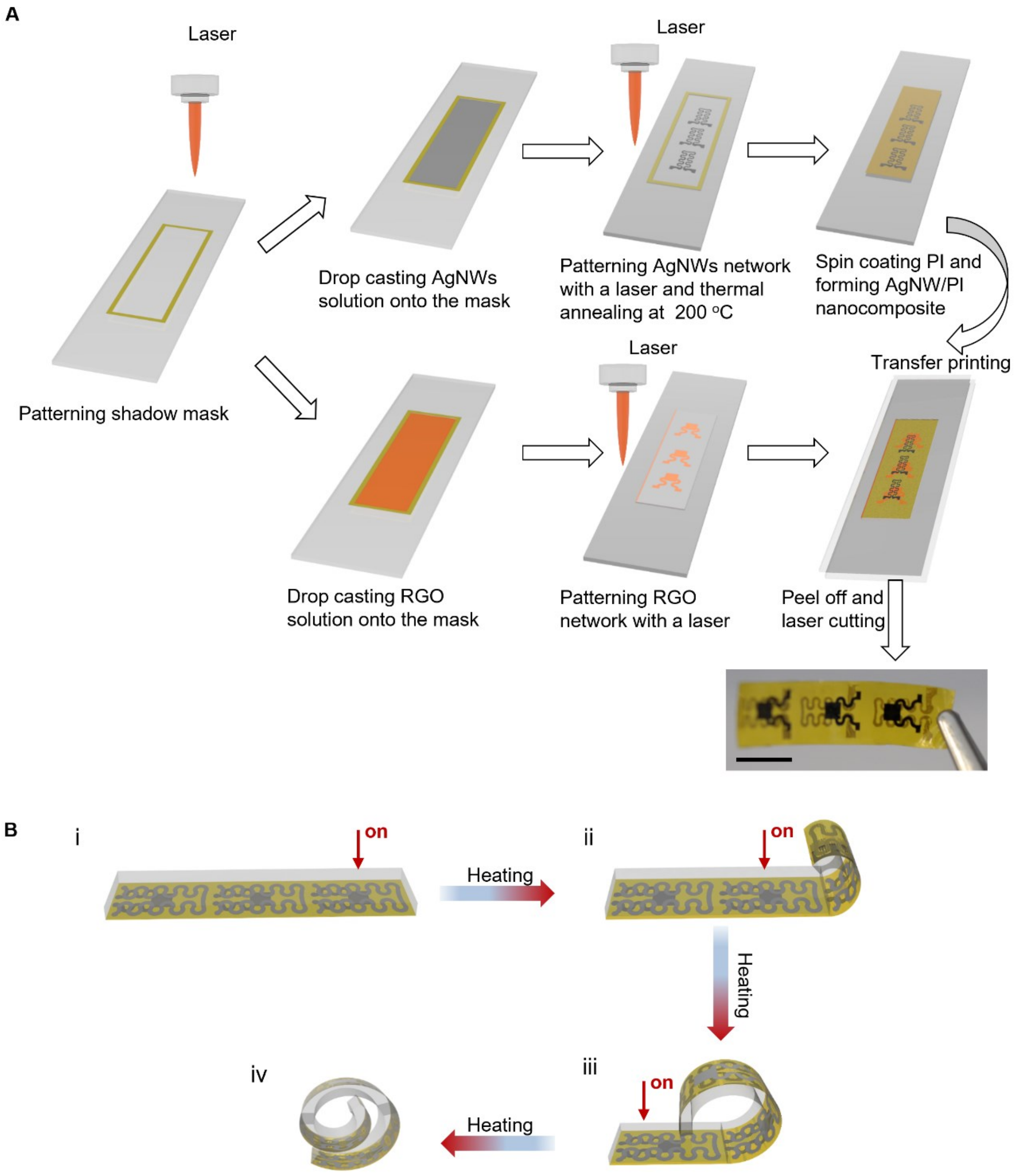

Fig. S23. Local and sequential control of a soft robotic finger. (**A**) Schematic illustration showing the fabrication process of a flexible, multifunctional film consisting AgNW/PI-based heaters that provide localized thermal activation, and RGO/PI nanocomposite temperature sensors that offer in-situ temperature monitoring. Scale bar, 10 mm. (**B**) Schematic illustration of the soft robotic finger to realize a programmable coiling motion in a stepwise fashion.

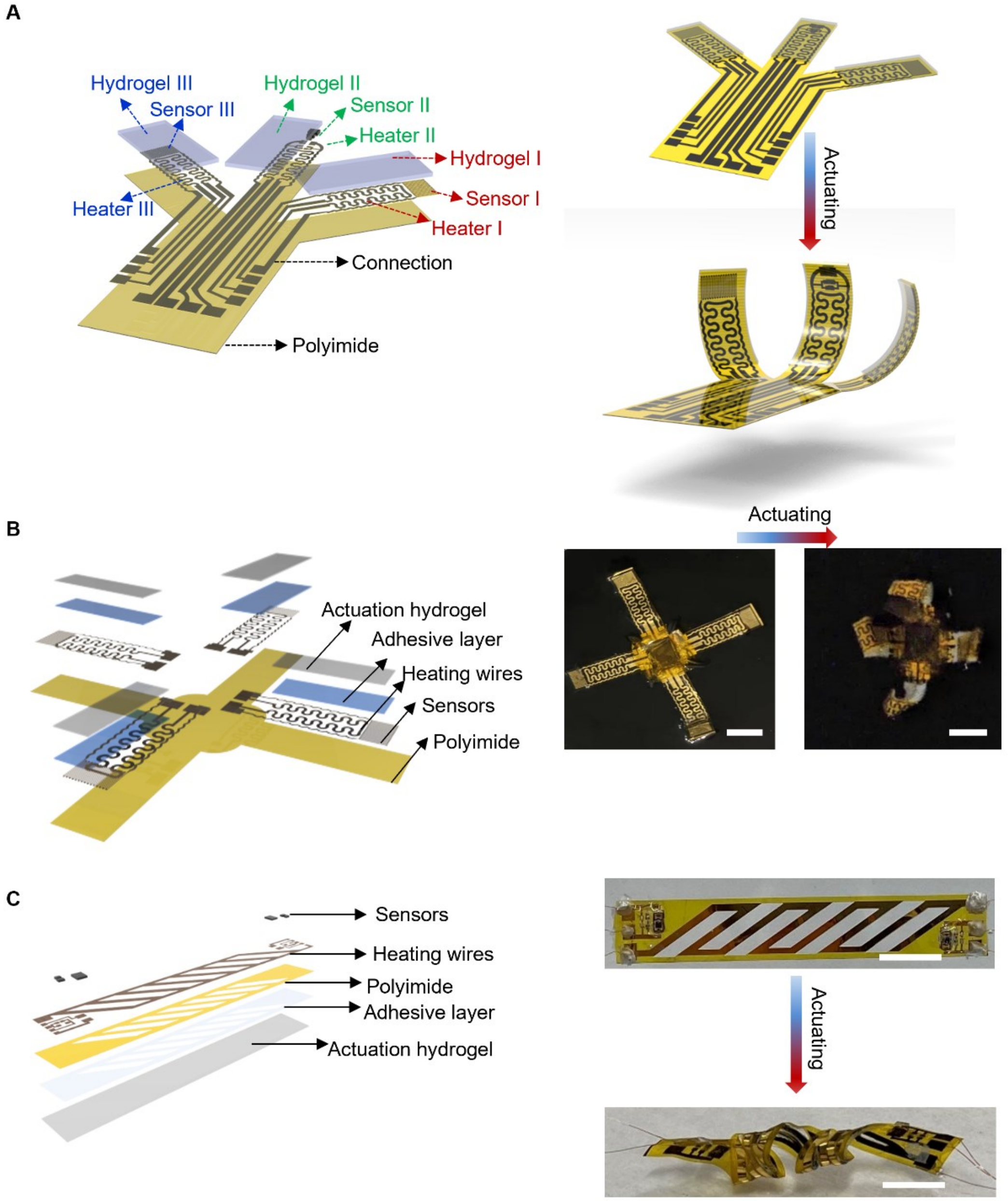

Fig. S24. Soft sensory robots with a broad range of motion configurations. (**A**) A soft robotic gripper with three sensory arms. (**B**) A soft starfish-like robot with four sensory arms. (**C**) A soft robotic cuff with optoelectronic sensors. Scale bars, 7 mm.

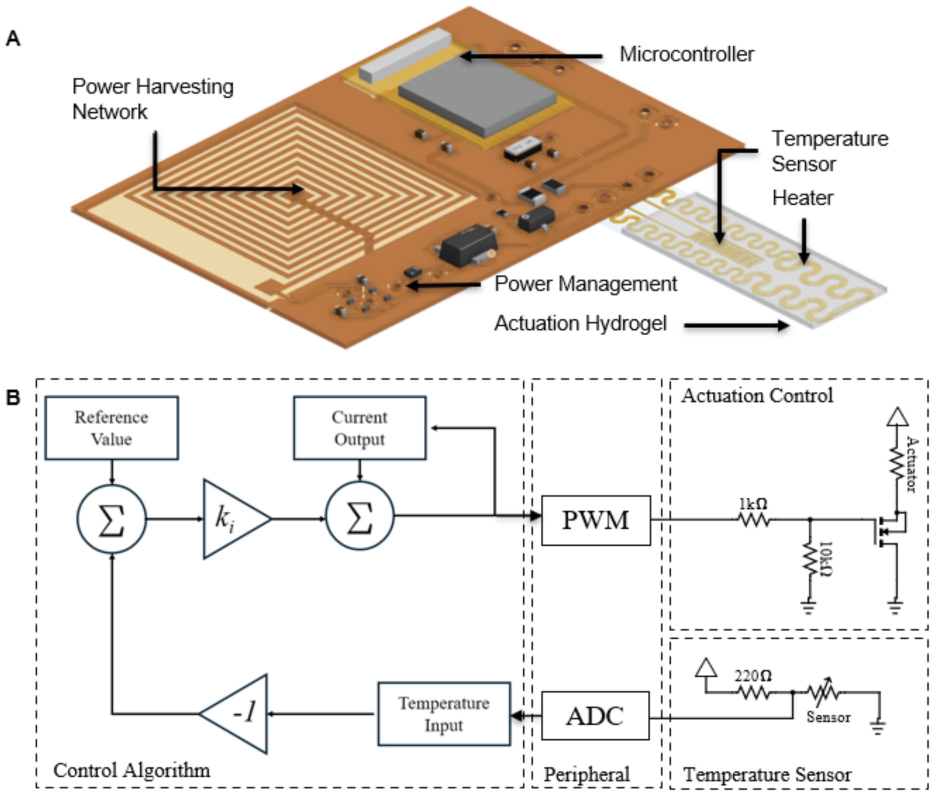

Fig. S25. Soft robots designed integrated sensing and actuation capabilities. (**A**) Schematic diagram of the responsive robotic gripper with control circuitry. (**B**) Functional block diagram of the algorithmic and hardware design of the adaptive control loop.

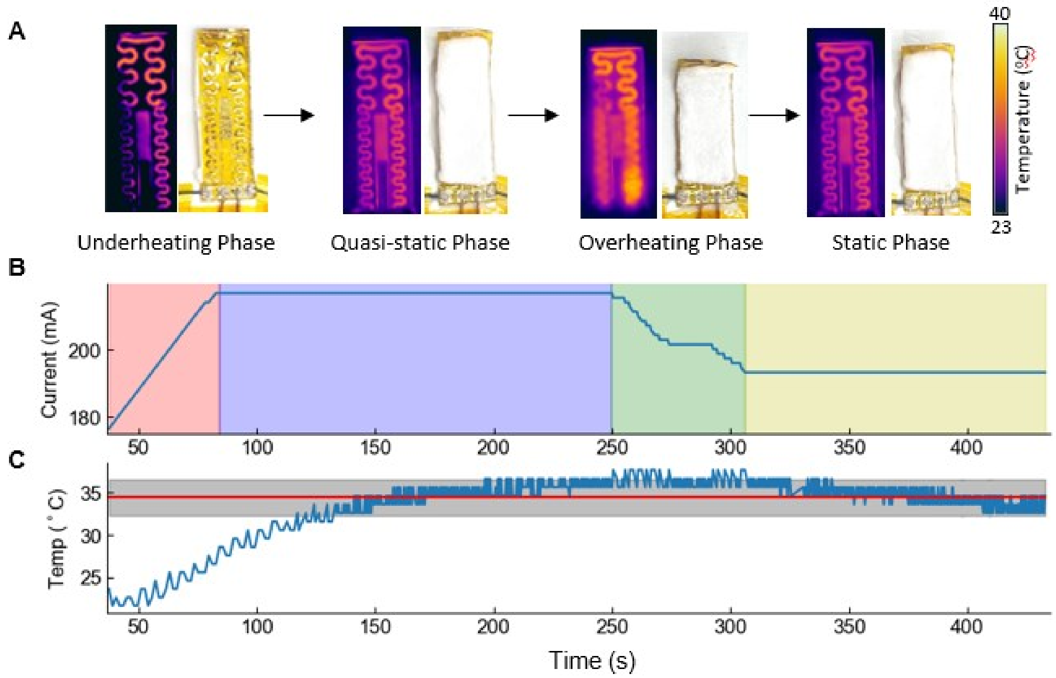

Fig. S26. Soft robots designed integrated sensing and actuation capabilities. (**A**) Infrared and optical images of the responsive robotic grippers at different stages of controlled actuation. (**B**&**C**) Time synchronized current (**B**) and temperature plot (**C**) capturing the controlled actuation changes after a sudden change in ambient temperature.

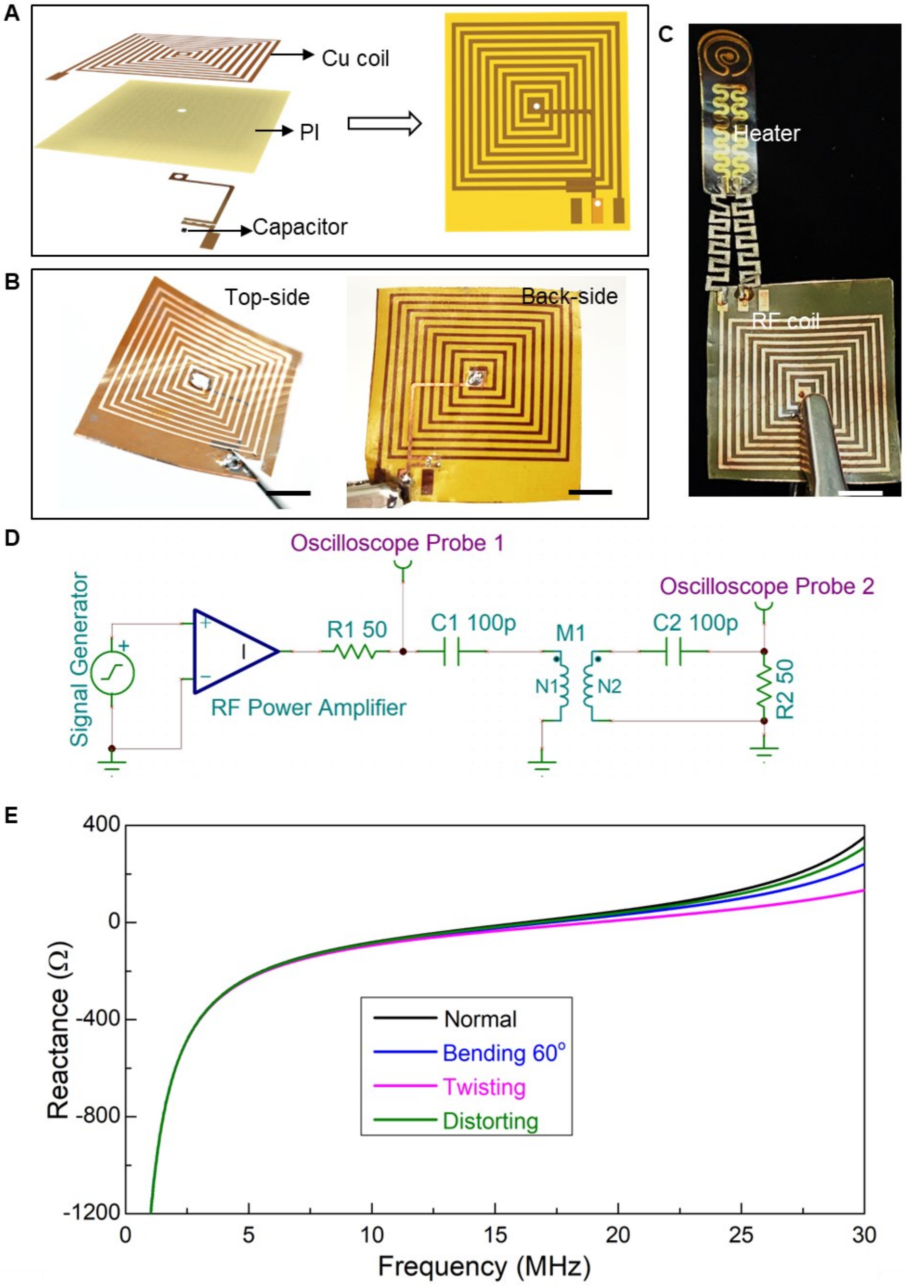

Fig. S27. Wireless power transfer system. (**A**) Schematic illustration of the receiver coil with a triple-layer structure. Here, the RF module is fully encapsulated with a conformal layer of parylene (thickness ~2 μm). (**B**) Optical images of the RF coil from the top and back sides. (**C**) Optical image of a single e-skin with circuits for wireless sensing and actuation. (**D**) The equivalent circuit diagram for the WPT system mainly includes: (i) RF generator producing an alternating current (AC) at a specific frequency; (ii) Transmitter coil with a tuning capacitor creating a resonant circuit; (iii) The receiver coil inducing an AC current due to the magnetic coupling with transmitter coil; (iv) The target electrothermal heater transfers the electrical energy to thermal energy. (**E**) The impedance variations of the RF coil under shape deformation at different frequencies. Scale bars, 5 mm.

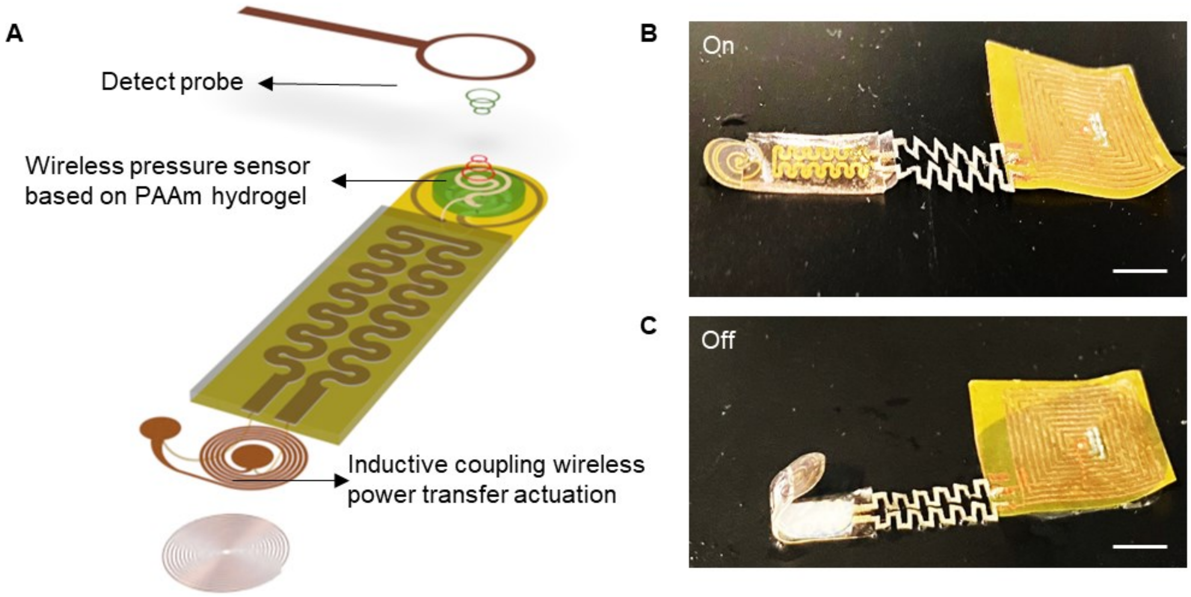

Fig. S28. Wireless sensing and actuation of a soft robotic finger. (**A**) Schematic illustration of the soft sensory robot consisting of wireless sensing and actuation sections. (**B&C**) Optical images of the soft robotic finger undergoing wireless actuation from a resting flat state (**B**) to a bent state (**C**) when wirelessly receiving actuation energy. Scale bars, 5 mm.

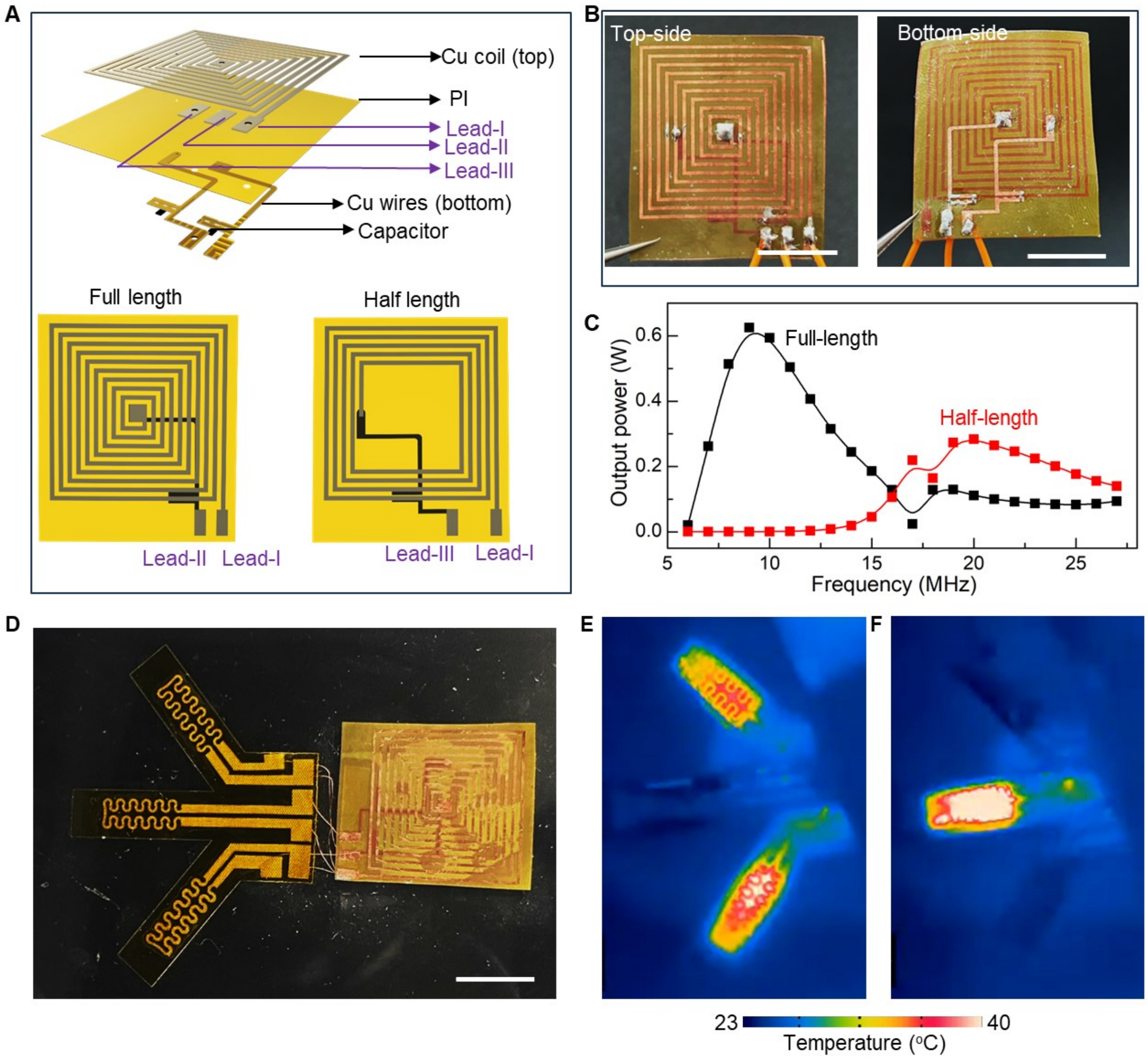

Fig. S29. Wireless locomotion control strategy for on-demand actuation of robotic grippers. (**A**) Schematic diagram of the LC frequency selection and power harvesting network. Here, the RF module is fully encapsulated with a conformal layer of parylene (thickness ~2 μm). (**B**) Optical images of the as-fabricated wireless harvesting network. (**C**) Experimental characterization of the resonance frequency and power transmission of different loads connected to the frequency selection and power harvesting network. (**D**) Optical image of the assembly of the robotic gripper and the wireless power harvesting network. (**E** and **F**)) Infrared images presenting the selective heating of the three-armed robotic gripper by employing distinct coupling frequencies. Scale bars, 5 mm.

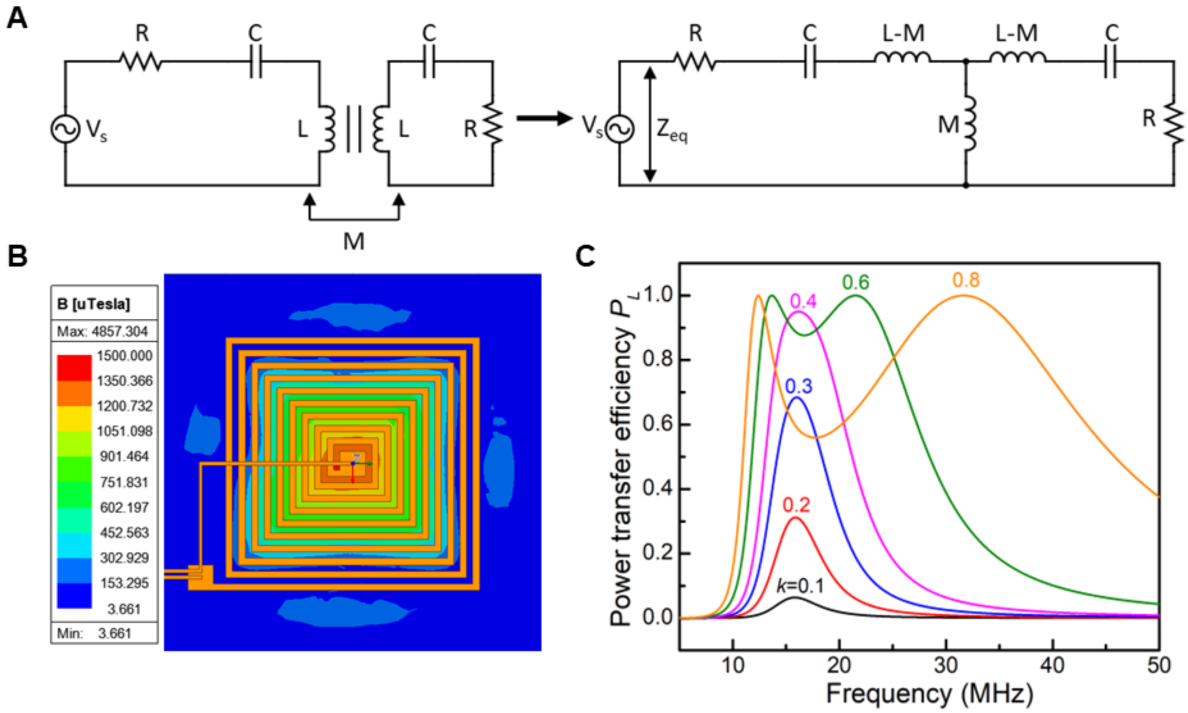

Fig. S30. Finite element analysis on wireless power transfer system (WPT). (**A**) The equivalent circuit of the WPT system. Left: The idealized circuit of the system. Right: The T-transformation of the equivalent circuit. (**B**) Magnetic field distribution of the pair of coils. (**C**) Numerical results of power transmission efficiency at different coupling coefficients *k*.

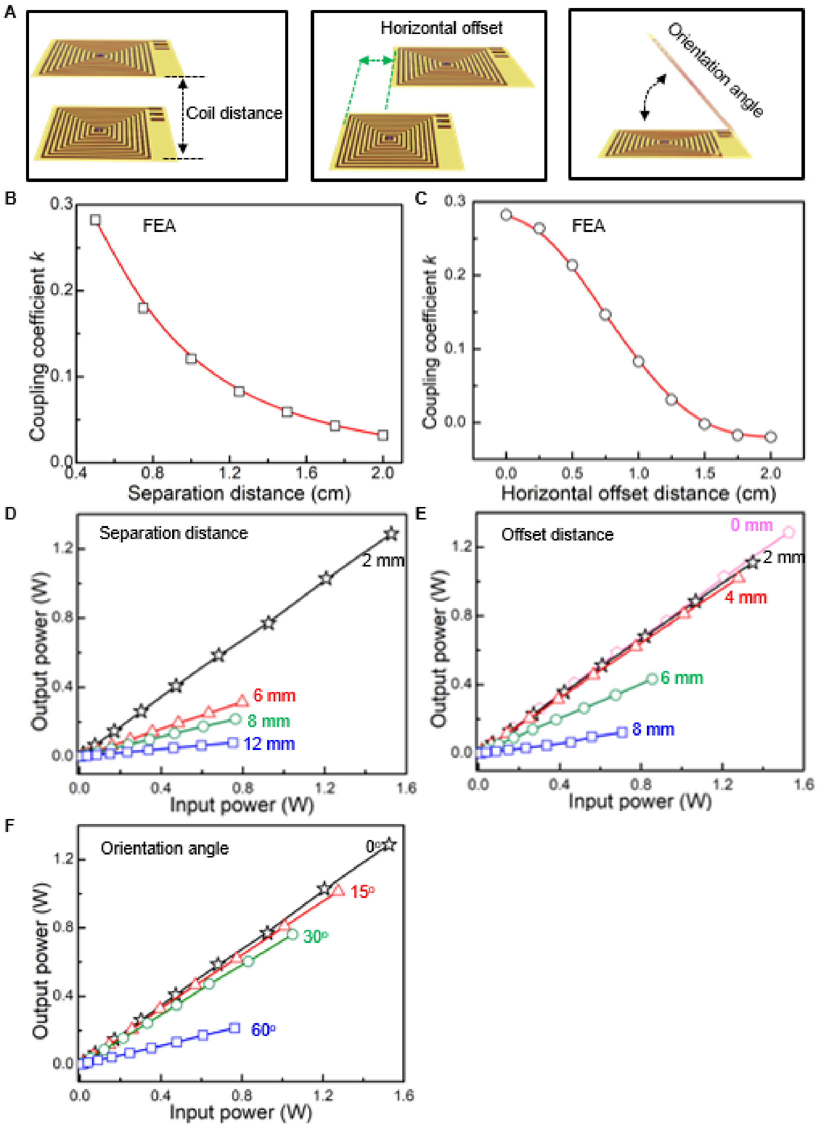

Fig. S31. The effect of separation distance, horizontal offset and orentation angle on the performance of WPT system. (**A**) Schematic illustrations of coil geometry and position in the WPT system. (**B** and **C**) FEA results showing the variations in the coupling coefficient of the WPT coils under different separation distances (**B**) and horizontal offsets (**C**). (**D-F**) Experimental evaluations of power transmission at resonance frequency (16 MHz) under different separation distances (**D**), horizontal offsets (**E**), and orientation angles (**F**).

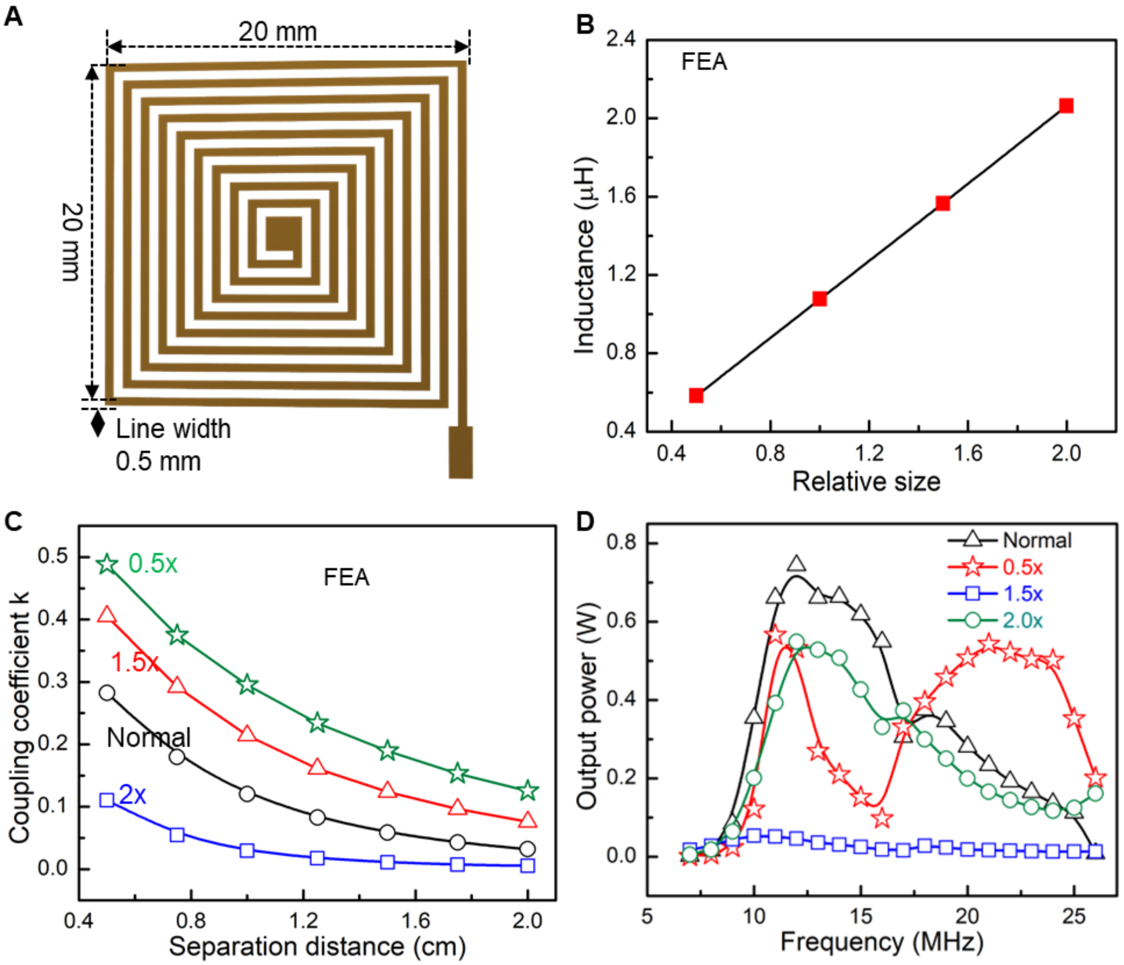

Fig. S32. The effect of coil size on the performace of WPT system. (**A**) Schematic illustration of a RF coil with a normal size. (**B** and **C**) FEA results of the self-inductance (**B**) and coupling coefficient (**C**) of the WPT coil with various sizes. Here, the relative size referes to the scaling ratio of enlarement or reduction compared to the normal size. (**D**) Experimental characterization of output power variations in relation to the frequency for RF coils with various sizes.

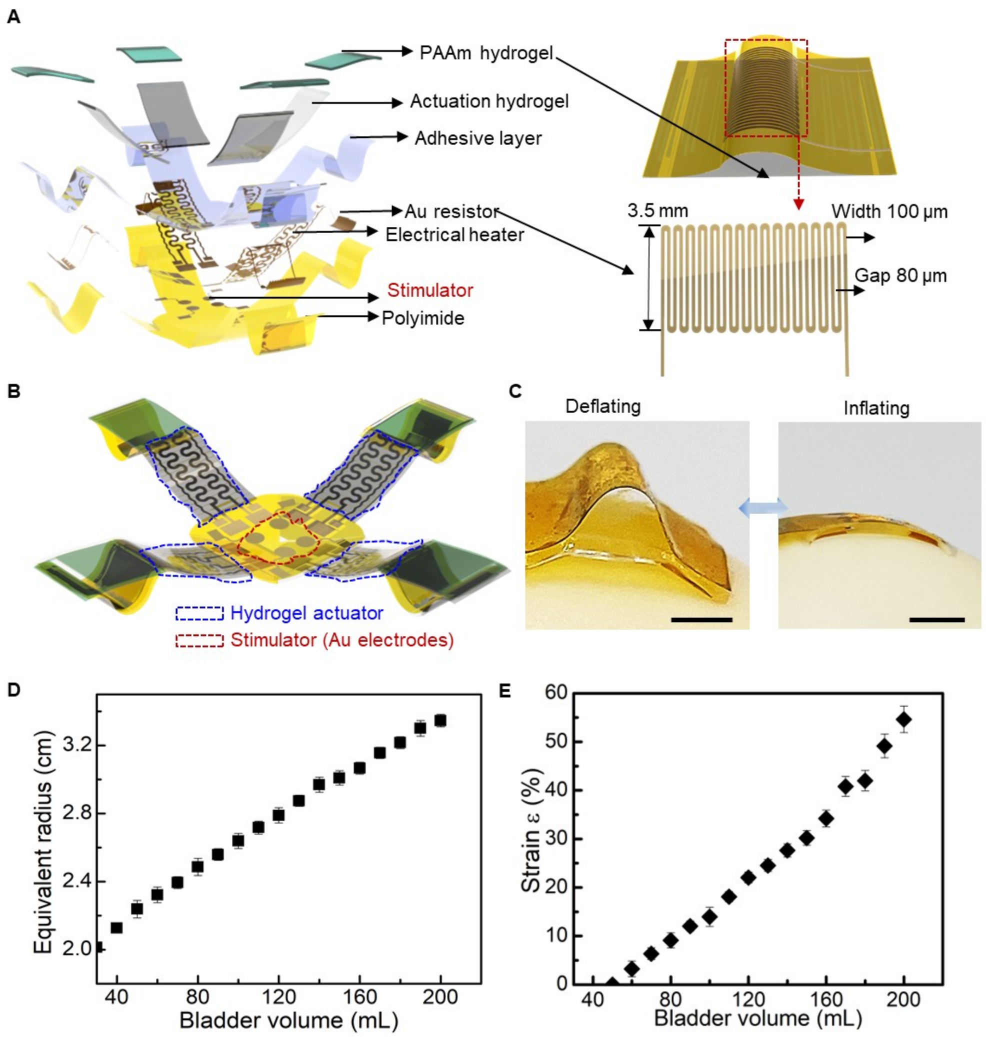

Fig. S33. Soft robotic gripper for monitoring bladder volume. (**A**) Schematic illustration showing exploded layout of the soft robotic gripper that incorporates actuator, sensor and stimulator components. The actuation component includes a passive layer made of an Au/PI bilayer with heat functionality, and an active layer of PNIPAM hydrogel. The sensing component includes an elastic poly(acrylamide) (~PAAm) hydrogel film and a serpentine Au/PI resistor to form a buckled strain sensor. The stimulator component is two pairs of electrical stimulation electrodes, employing Au for its exceptional electrical conductivity, biocompatibility, and stability in biological settings. (**B**) Detailed schematic illustration for the soft robotic finger, highlighting the direct tissue contact by the stimulation electrode (indicated with a red dashed line). Here the hydrogel actuators are intentionally positioned on the device's arms, marked by the blue lines, ensuring that they do not hinder the electrodes' direct engagement with the tissue, facilitating efficient electrical stimulation. (**C**) Optical images of a 3D buckled strain sensor attached to an artificial bladder in an inflated and deflated state, respectively. Scale bars, 3 mm. (**D**) Measured equivalent radius as a function of volume of the artificial bladder. Urine filling inside the bladder increases its equivalent radius. (**E**) Results of strain change measured from the soft robotic gripper as a function of bladder volume.

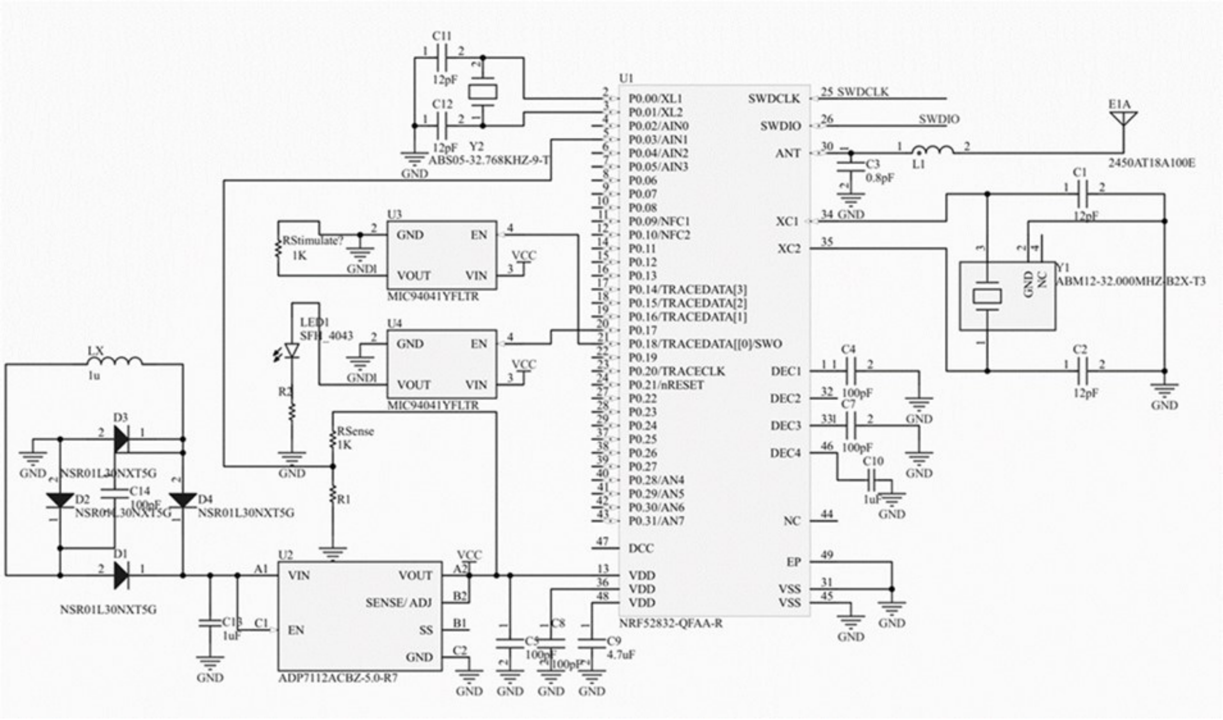

Fig. S34. Circuit diagram of the signal conditioning circuitry.

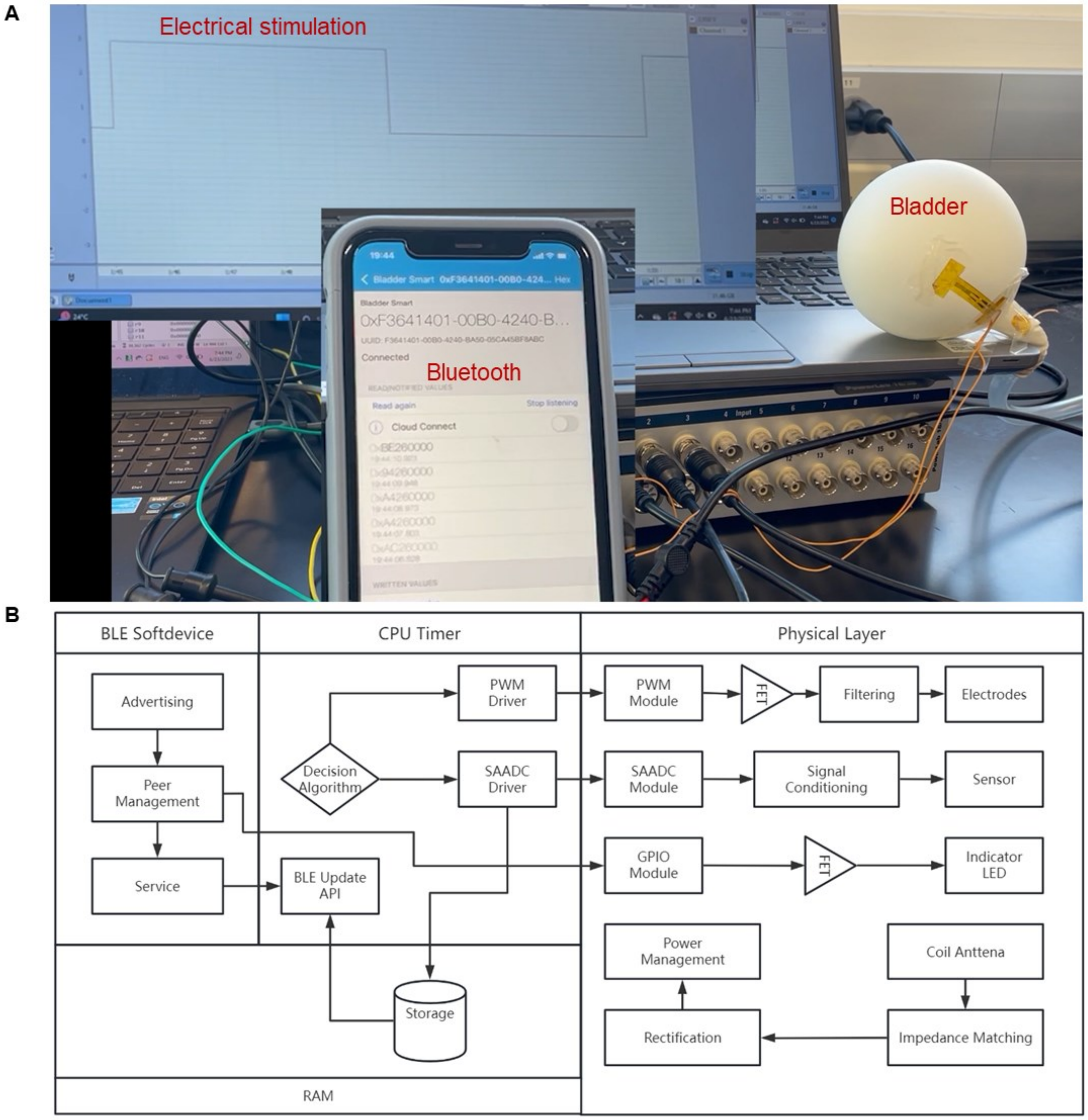

Fig. S35. *In vitro* demonstration of the integrated bladder system. (**A**) A photograph showcasing a balloon biomimicking bladder integrated with a wireless closed-loop system including a signal conditioning circuit for strain sensors, a Bluetooth System-on-Chip, and a MOSFETs for amplification of electrical stimulation. Here, the system allows for inflation and deflation of a balloon to simulate the storage and release states of a bladder. (**B**) The flow chart of the wireless closed-loop controlled bladder electrical stimulation module.

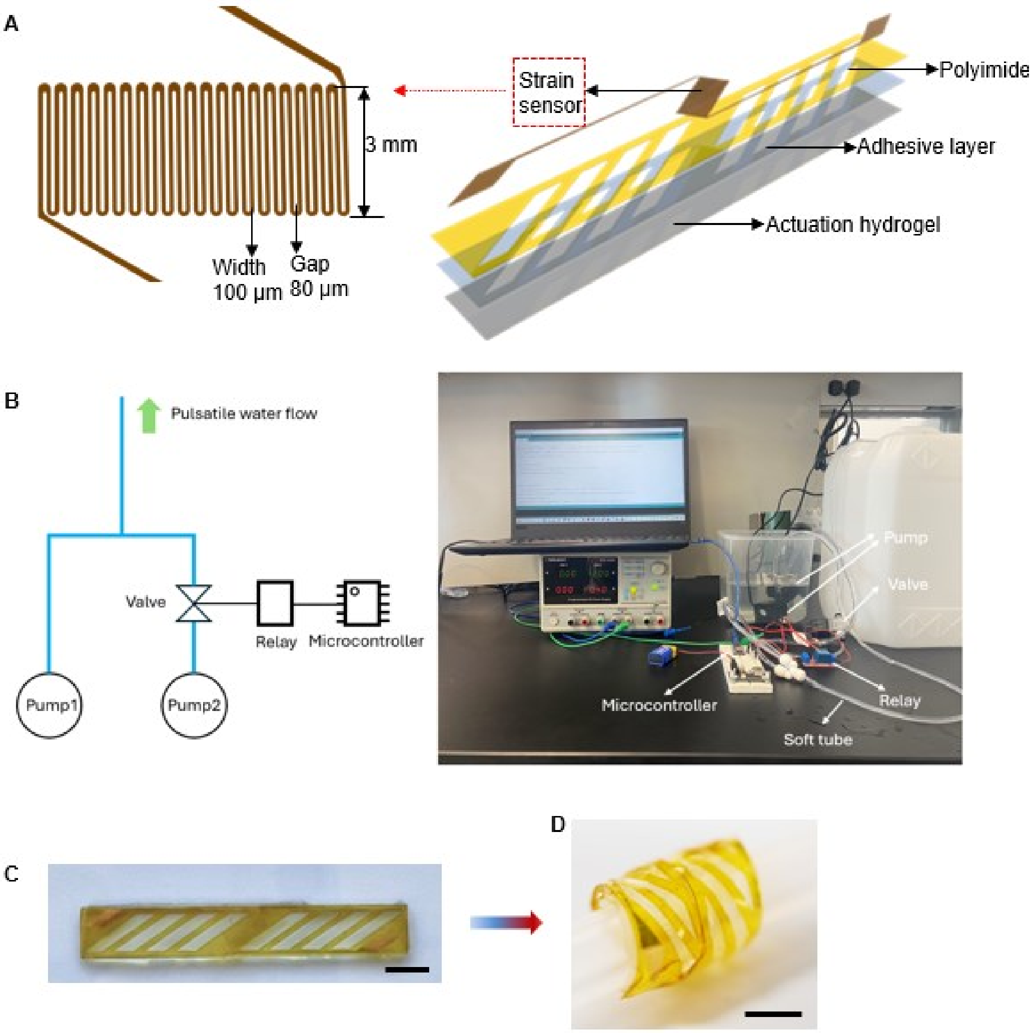

Fig. S36. A soft robotic cuff for real-time monitoring of blood pressure. (**A**) Schematic illustration of an exploded view on the soft robotic cuff. The system consists of an actuator based on a PNIPAM-based bilayer structure and a strain sensor made of a serpentine Au/PI resistor. (**B**) *In vitro* setup for artificial artery model. (**C**) Optical image of the soft robotic cuff in a standby flat state. (**D**) The soft robotic cuff wraps around an artificial blood vessel upon thermal actuation. scale bars, 7 mm.

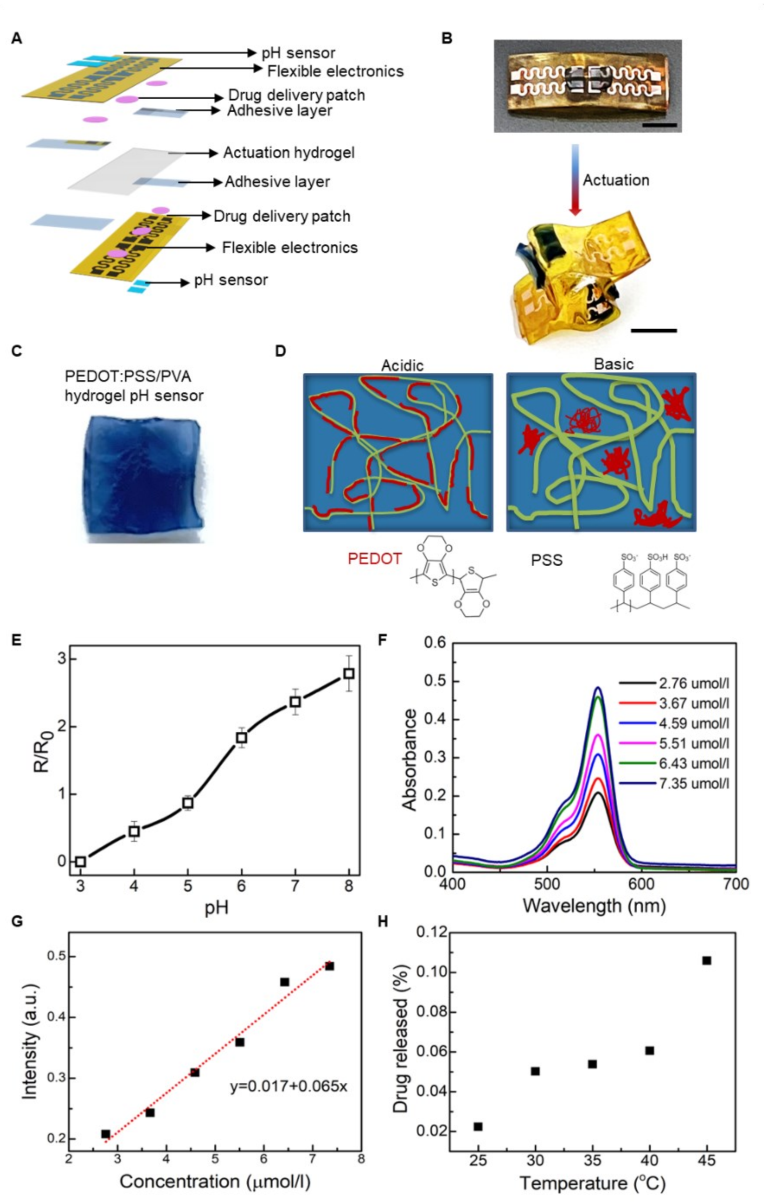

Fig. S37. A soft ingestible robot for continuous, long-term monitoring of gastric pH and targeted drug delivery inside the stomach. (**A**) Schematic illustration of the exploded layout of the soft robot, showing an actuation layer (bilayers of PNIPAM/Au-pattern PI), a drug-releasing layer (composite of PLGA and rhodamine-B patch), and a pH sensing layer (PEDOT:PSS/PVA hydrogel). (**B**) Optical images of the soft robot undergoing a structural transformation from a flat state to an arch state. Scale bars, 5 mm. (**C**) Optical image of a PEDOT:PSS/PVA hydrogel for forming a pH sensor. (**D**) Schematic illustration of the impact of pH change on the molecular reconfiguration of PEDOT:PSS. (**E**) Measured correlation between resistance and environmental pH for the PEDOT:PSS/PVA hydrogel. (**F**) Measured UV-vis absorbance spectrum of the rhodamine-B solution with various concentrations in PBS solution. (**G**) Calculated calibration curve of rhodamine-B based on the UV-vis spectral analysis. (**H**) 1 h release of model drug (rhodamine B) from PLGA matrix under various temperatures.

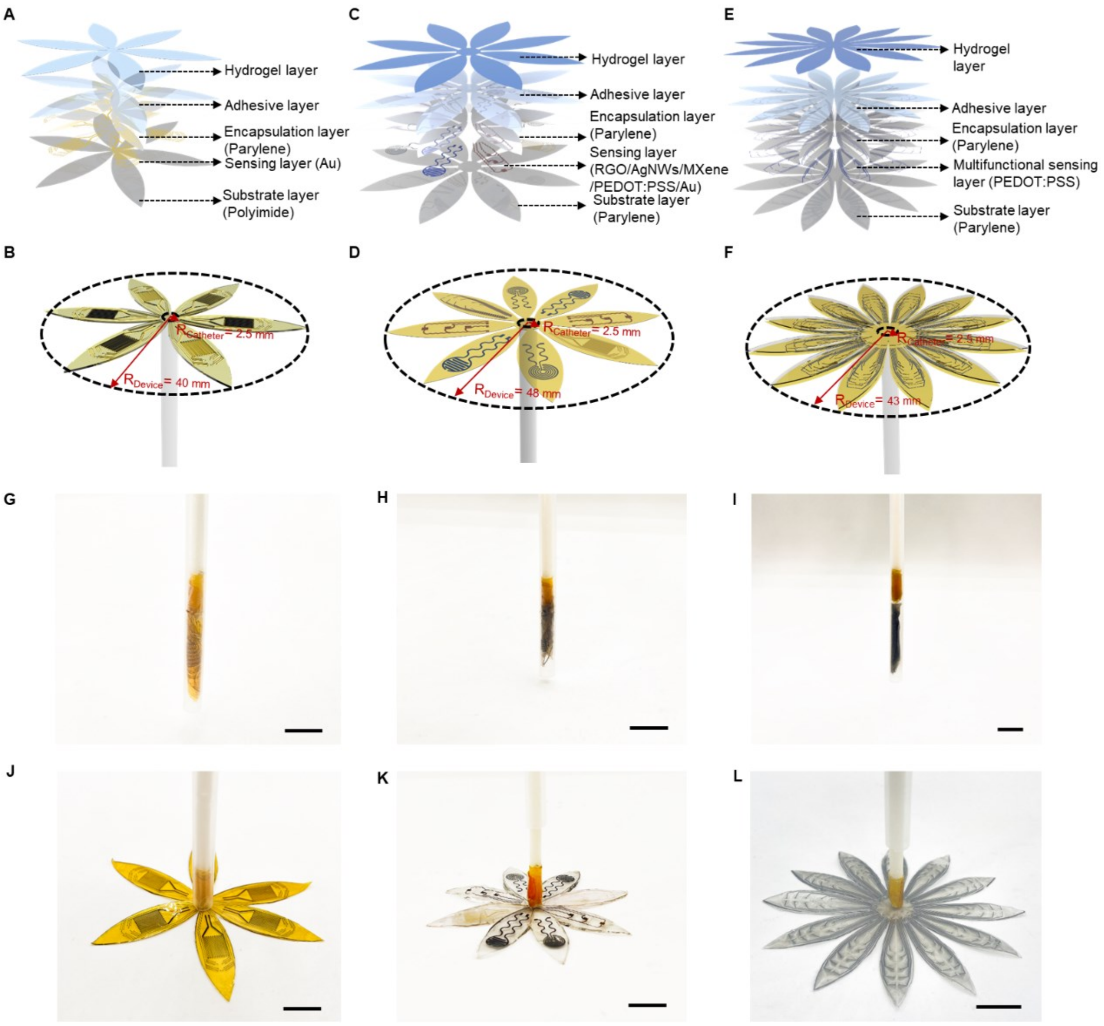

Fig. S38. Illustration of a starfish-like implant exhibiting significant modularity and adaptability. (**A-F**) Representation of a starfish device featuring six arms (**A**&**B**), eight arms (**C**&**D**), and twelve arms (**E**&**F**). (**A**, **C**, and **E**) Exploded views of the devices, showcasing they are primarily composed of 3 layers, including a hydrogel actuation layer (thickness ~300 μm), an adhesive layer, and an e-skin layer comprising functioning units based on materials such as Au, AgNWs, PEDOT:PSS, RGO, and MXene. The e-skin layer has the following respective thicknesses: the encapsulation layer is ~2 μm, the Au-based sensing layer is ~200 nm, the RGO/MXene/AgNWs/PEDOT:PSS-based layer is ~2 μm, and the substrate is ~10 μm. Notably, we can integrate diverse sensing units onto the e-skin layer for specific applications, enhancing functionality. Simultaneously, the encapsulation layer ensures prolonged biocompatibility, crucial for implantable devices. (**B**, **D**, and **F**) The varying diameters of the catheter and device, highlighting the adjustable aspect ratio of relative projection area between the device and catheter, such as ~256 (**B**), ~380 (**D**), and ~295 (**F**). (**G-L**) The devices show the capability to be securely accommodated in a catheter (**G-I**), and subsequently deployed from it (**J-L**), showcasing the operational flexibility. Scale bars, 1 cm.

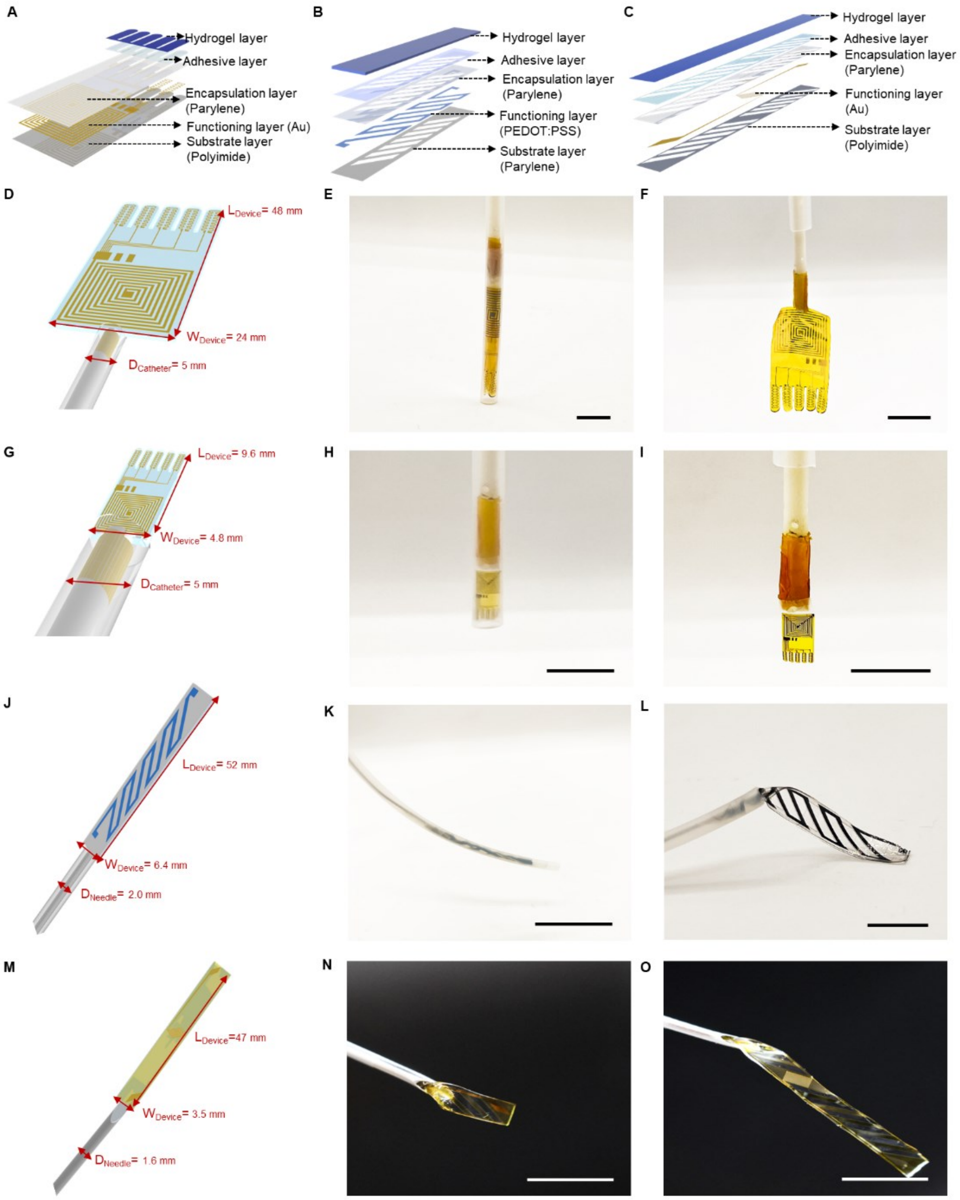

Fig. S39. Illustration of a diverse range of devices, exhibiting versatility in both shape and dimensions. (**A-C**) Exploded views highlight the device components: a hydrogel actuation layer (thickness ~300 μm), an adhesive layer, and an e-skin layer featuring functioning units fabricated with Au (thickness ~200 nm) and PEDOT:PSS (thickness ~2 μm). Notably, the e-skin layer is encapsulated by a parylene layer (thickness ~2 μm) to ensure sufficient biocompatibility. (**D**, **G**, **J**, and **M**) Schematic illustrations display the dimensions of both the device and the delivering tools, highlighting the broad range of aspect ratios of relative projection area between them, as exemplified by ~58 (**D**), ~2 (**G**), ~106 (**J**), and ~82 (**M**). (**E**, **F**, **H**, **I**, **K**, **N** and **O**) Optical images depict that soft robotic devices can be efficiently housed in and released from a catheter (**E**, **F**, **H**, and **I**) or even a 14-gauge Tuohy needle (**N**&**O**), demonstrating the versatility in delivery methods for various applications. Scale Bars, 1 cm.

Table S2 Aspect ratio comparison of different minimally invasive implants.

Representative examples	Device Dimensions (mm)	Incision size/catheter diameter (mm)	Device area (mm^2)	Incision/catheter area (mm^2)	Area ratio	Ref.
An electrocorticography system	Starfish structure with six legs Diameter~40	20	1256	314	4	[104]
Spinal cord stimulation electronics	2D device Length~54 Width~14	1.6	756	2	378	[105]
Therapeutic epicardial device	2D device Width~4	1.6	16	2	8	[106]
Peripheral nerve stimulation magnetoelectric implant	Bulk device Length~9 Width~2	3	18	7	2.6	[107]
Syringe-injectable electronics	2D mesh device Width~5	0.25~0.45	25	0.05~0.16	500~156	[108]
Flexible neural implant	2D device Width~0.18 Length~5	0.8	0.9	0.5	1.8	[109]
Shape-memory cardiac patch	2D mesh device Width~10	1	100	0.8	125	[110]
Syringe-injected brain probe	Cylinder Diameter ~0.1 Height~0.8	0.1~0.2	Lateral surface area ~0.25	0.008~0.03	31.4~8.4	[111]
Skin-inspired sensory robots	Starfish structure with eight legs Diameter~96	5	7235	19	380	This work

Note: The aspect ratios of devices in our work are adjustable and can span a broad spectrum, allowing adaptability for a variety of medical applications.

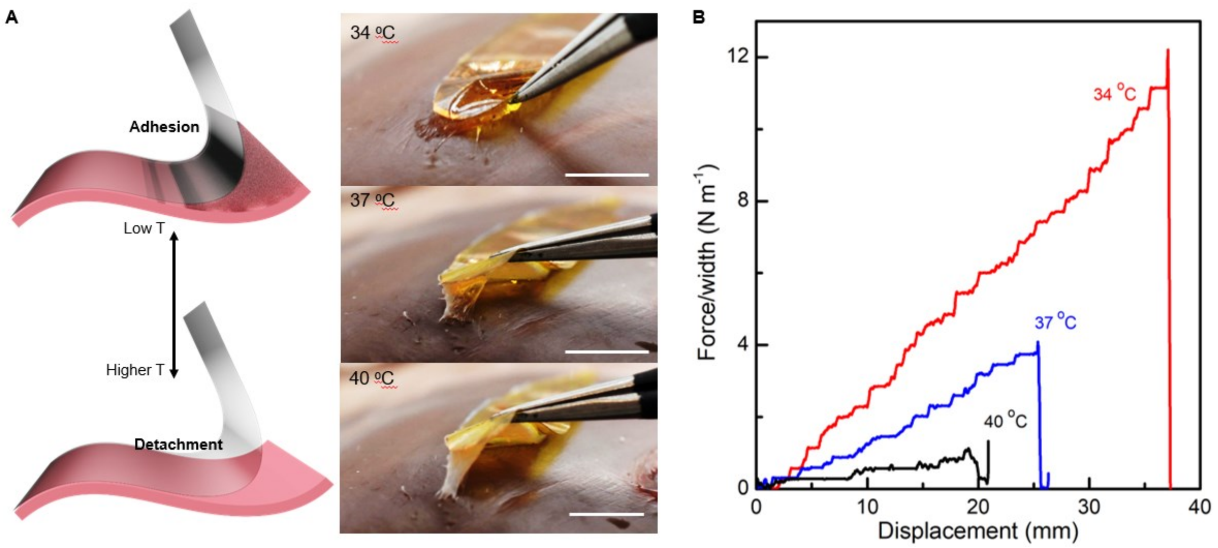

Fig. S40. Demonstration of hydrogel adhesion to various biological surfaces. (**A**&**B**) Photographs (**A**) and measured adhesion force (**B**) of a PNIPAM-*co*-PAAm hydrogel with an LCST of 36 ºC across different temperatures. Below the LCST, the hydrogel exhibits robust adhesion below its LCST, which gradually loses above this threshold. Scale bars, 5 mm.

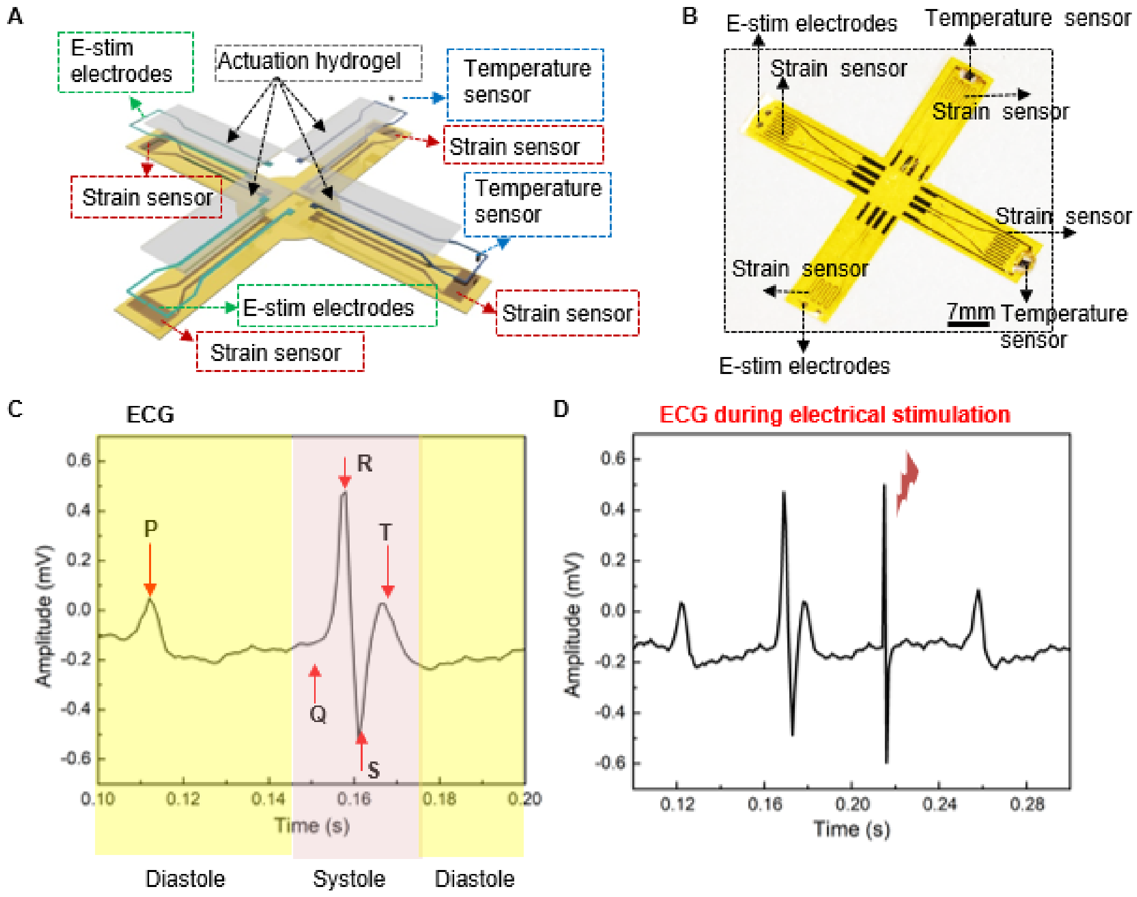

Fig. S41. A soft robotic thera-gripper with four arms for epicardial sensing and stimulation. (**A**) An exploded schematic illustration of the soft robotic thera-gripper consisting of actuators based on actuation hydrogel, four strain sensors made of serpentine Au/PI resistors, two temperature sensors made of thermal resistors, and two electrical stimulation (E-stim) electrodes made of Au. The use of Au ensures superior electrical conductivity, biocompatibility, and stability within biological environments, guaranteeing reliable and precise electrical stimulation essential for effective therapeutic interventions. (**B**) Optical image of a flexible electronic film serving as the sensory component for the soft robotic thera-gripper. (**C**) The surface ECG trace without electrical stimulating. (**D**) The surface ECG trace during electrical stimulation using a pair of Au E-stim electrodes (pulse duration = 1 ms, pulse amplitude = 500 mV, cycle length = 377 ms).

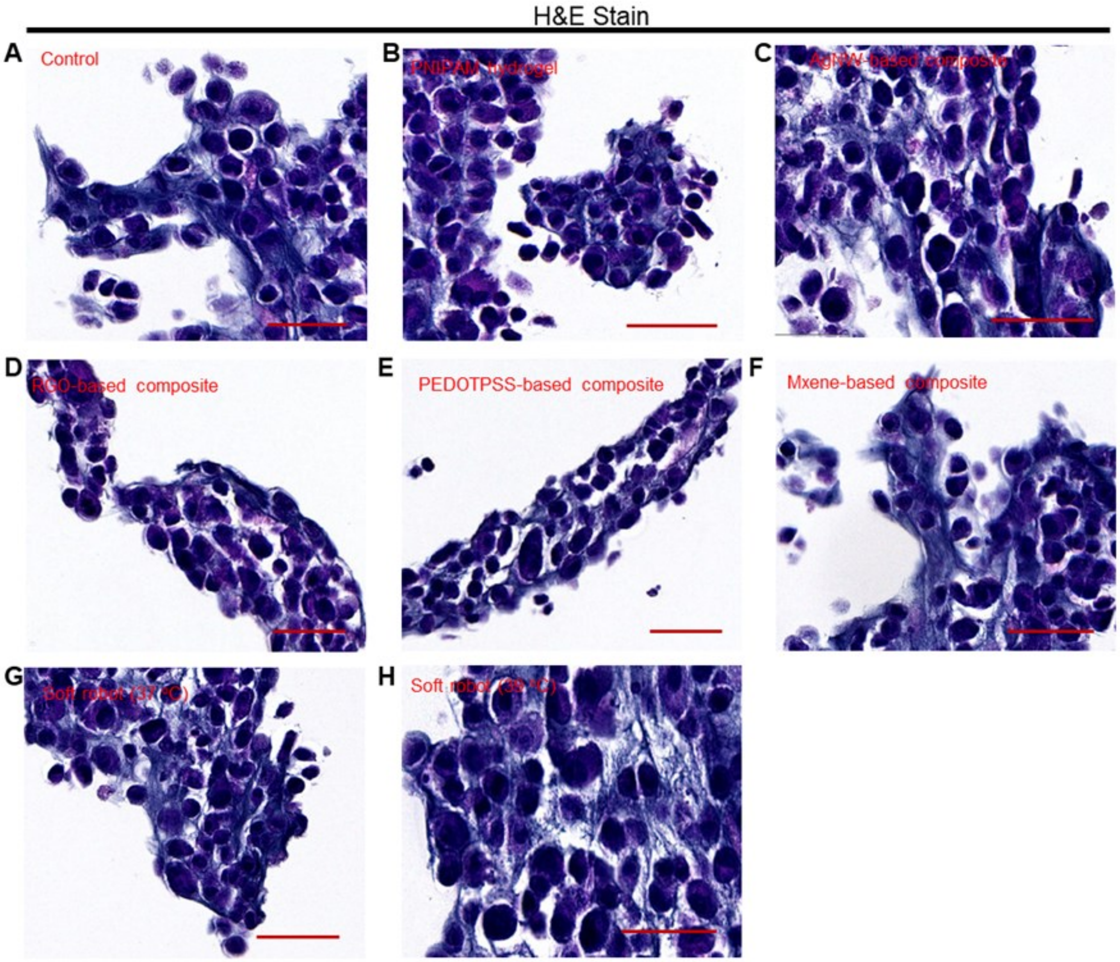

Fig. S42. Cell viability assessment of soft robotic implants. (**A**-**H**) Confocal microscope images of 3T3-J-2 cells before (control) and after exposure to various functional nanocomposites, including PNIPAM hydrogel, AgNWs, RGO, PEDOT:PSS and MXene-based composites, as well as as-prepared soft robotic devices integrated with an e-skin layer and PNIPAM hydrogel-muscle layer, incubated for 48 hours. (**A**) Control. (**B**) Pure PNIPAM hydrogel. (**C**) AgNW-based composite. (**D**) RGO-based composite. (**E**) PEDOT:PSS-based composite. (**F**) MXene-based composite. (**G** & **H**) The as-prepared soft robot integrated with the aforementioned e-skin layer and hydrogel muscle layer, incubated at 37 °C (**G**), and 39 °C (**H**), respectively. Hematoxylin & eosin (H&E) staining. Scale bars, 50 μm.

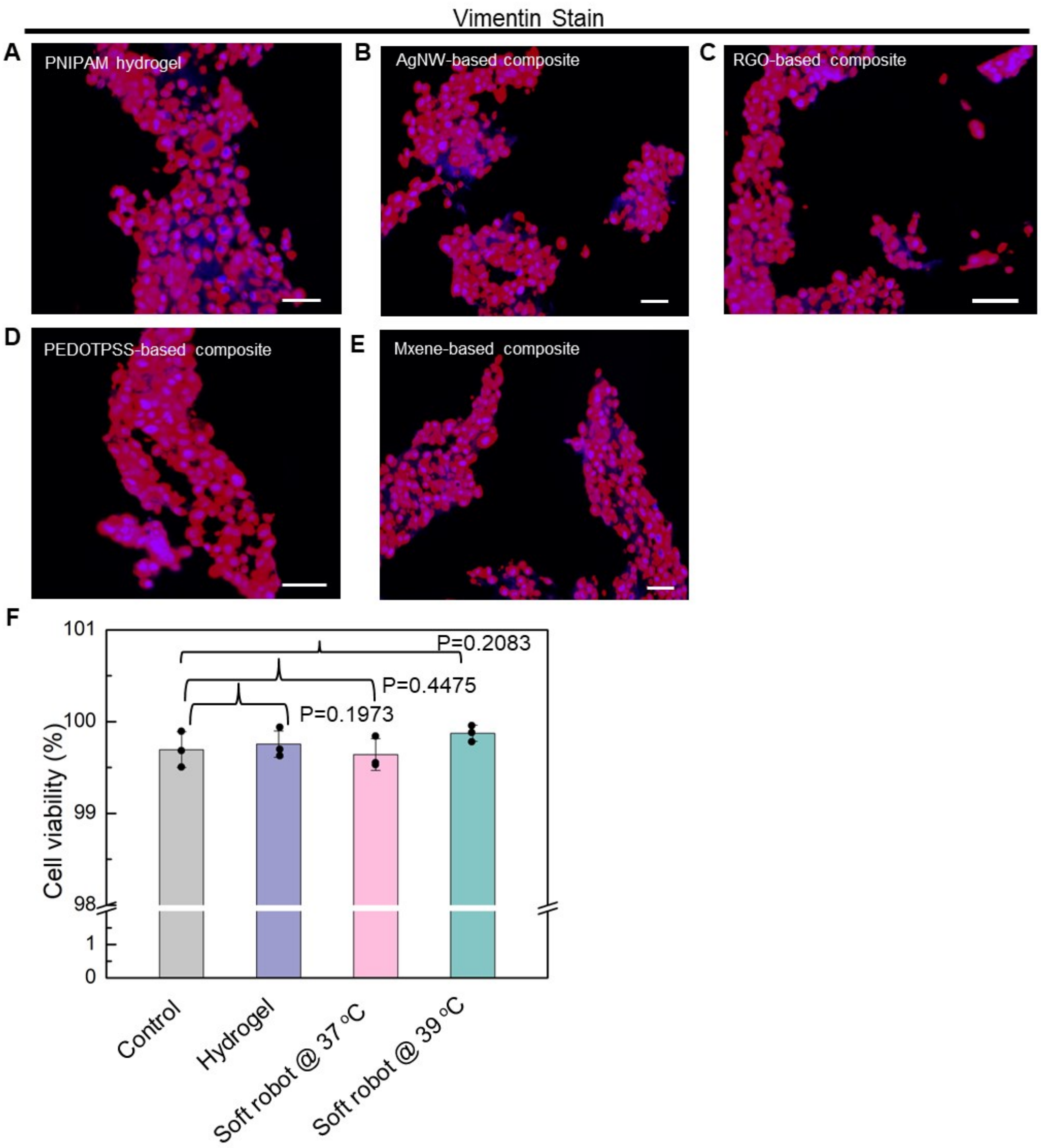

Fig. S43. Cell viability assessment of soft robotic implants. (**A**-**E**) Confocal microscope images of 3T3-J-2 cells exposure to various functional nanocomposites, including PNIPAM hydrogel, AgNWs, RGO, PEDOT:PSS and MXene-based composites, incubated for 48 hours. (**A**) Pure PNIPAM hydrogel. (**B**) AgNW-based composite. (**C**) RGO-based composite. (**D**) PEDOT:PSS-based composite. (**E**) MXene-based composite. (**F**) Comparative cell viability before and after soft robot's exposure, indicating that 3T3-J-2 cells exposed to as-designed soft robots have no decreased viability. Mean ± S.D. *n*=3. *P* value by Unpaired *t*-test. Scale bars, 50 μm.

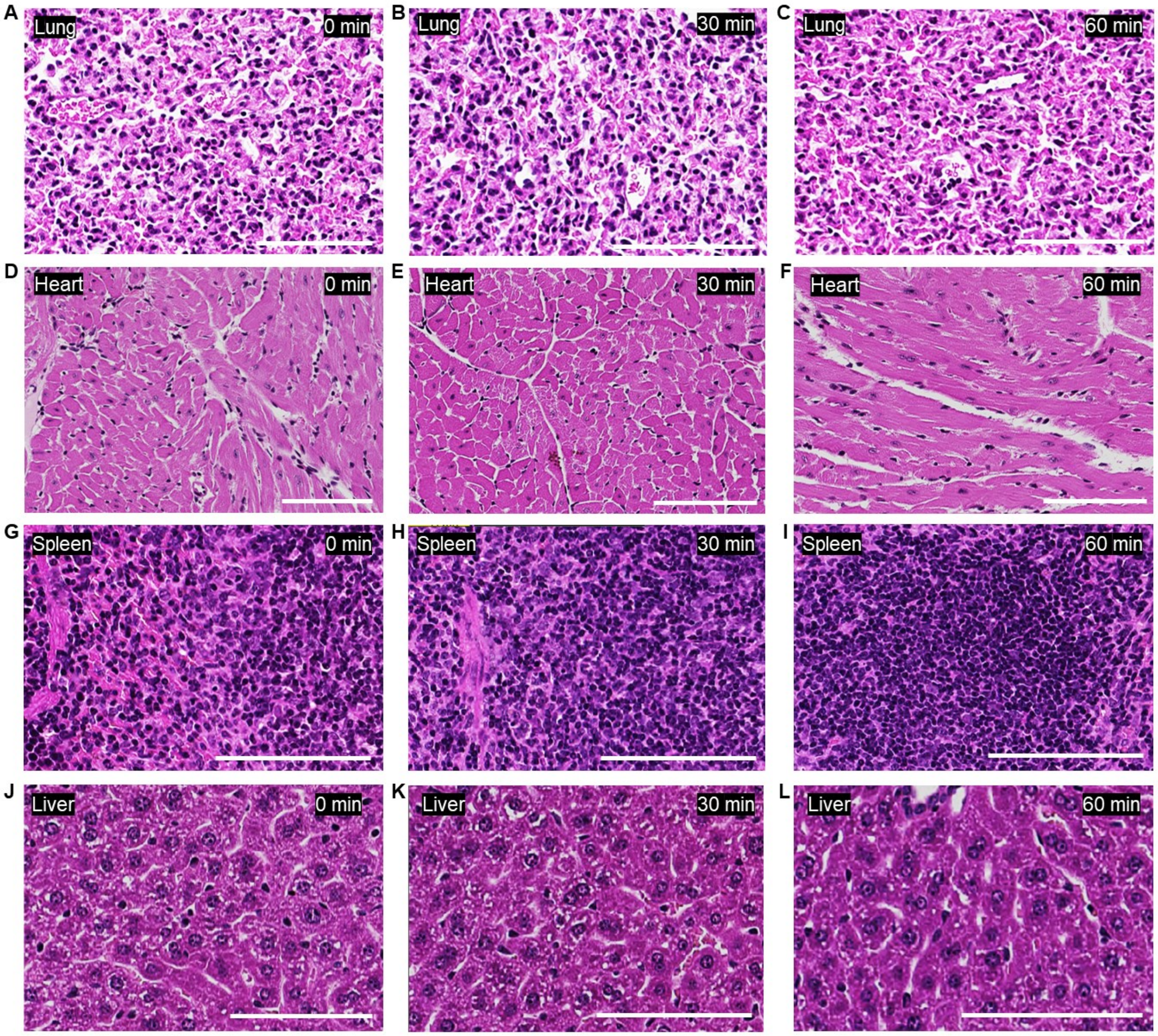

Fig. S44. Histological analysis (H&E staining) of biological tissues after thermal stimulation using a robotic implant. Histological analysis of lung tissue (**A**-**C**), heart tissue (**D**-**F**), spleen tissue (**G**-**I**), and liver tissue (**J**-**L**) after thermal stimulation at a power of 0.1 W, with durations of half an hour and one hour, respectively. (H&E staining, N=3). Scale bars, 100 μm.

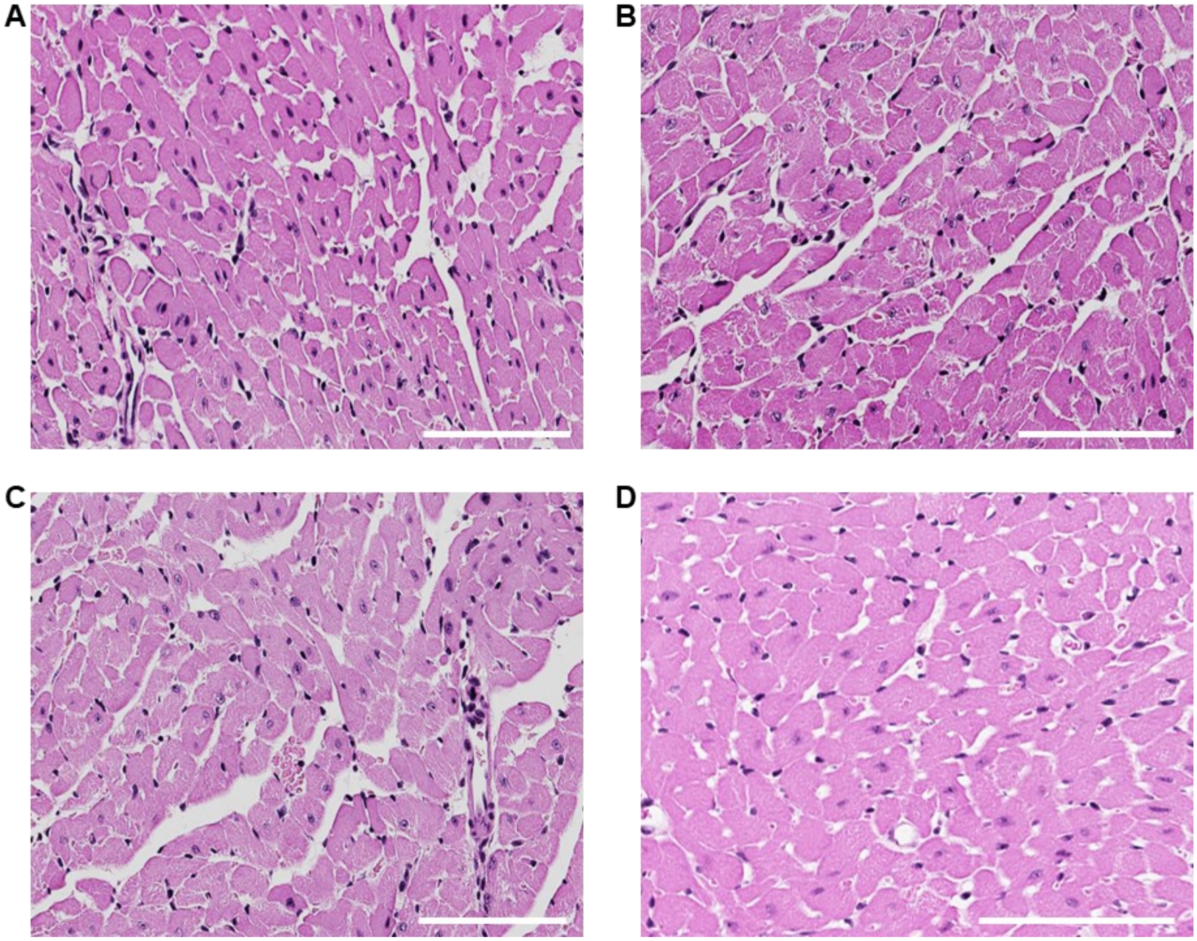

Fig. S45. Histological analysis (H&E staining) of the cardiac tissue after implantation of a hydrogel-based thera-gripper. (**A**) Day 1. (**B**) Day 3. (**C**) Day 7. (**D**) Day 14. (H&E staining, N=3). Scale bars, 100 μm.

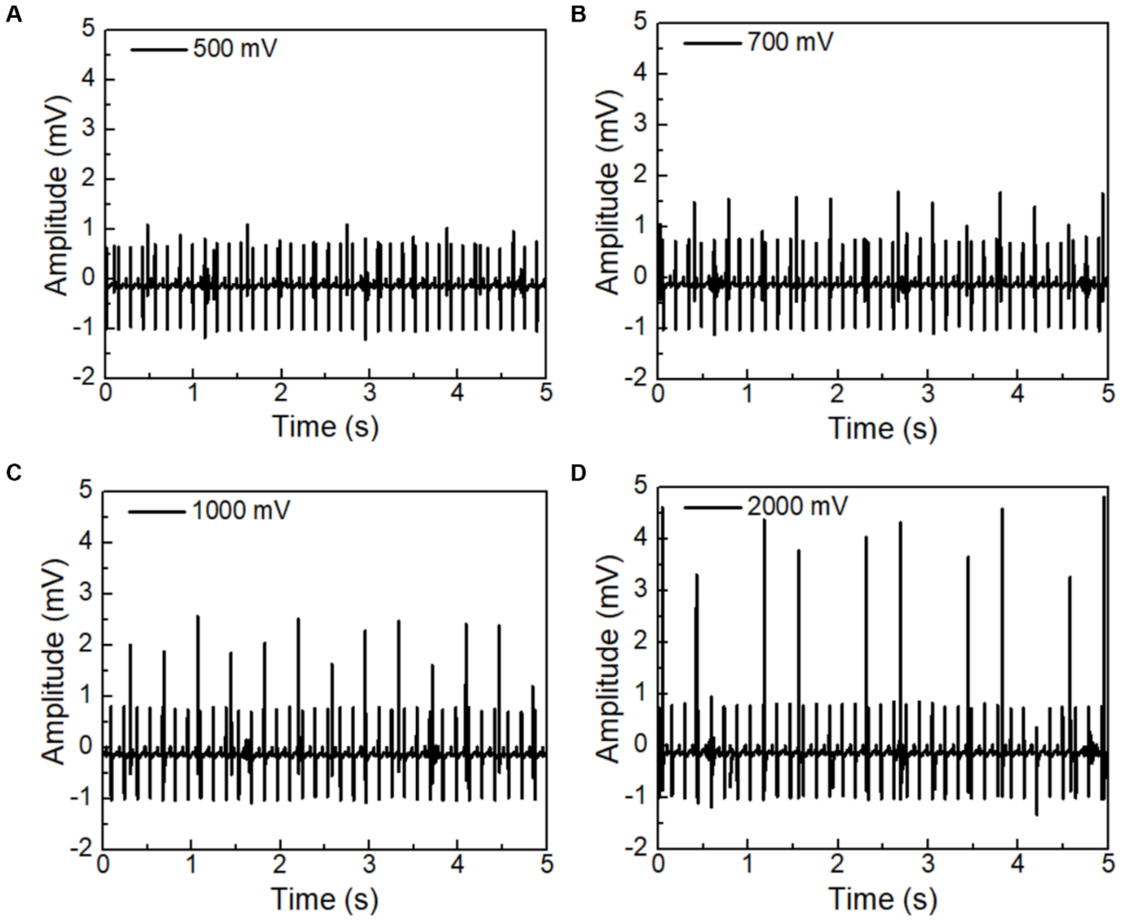

Fig. S46. Representative voltage traces of the ECG under electrical stimulation with various voltages ranging from 500 mV to 2000 mV with 1ms width at 2.65 Hz. (**A**) 500 mV. (**B**) 700 mV. (**C**) 1000 mV. (**D**) 2000 mV.

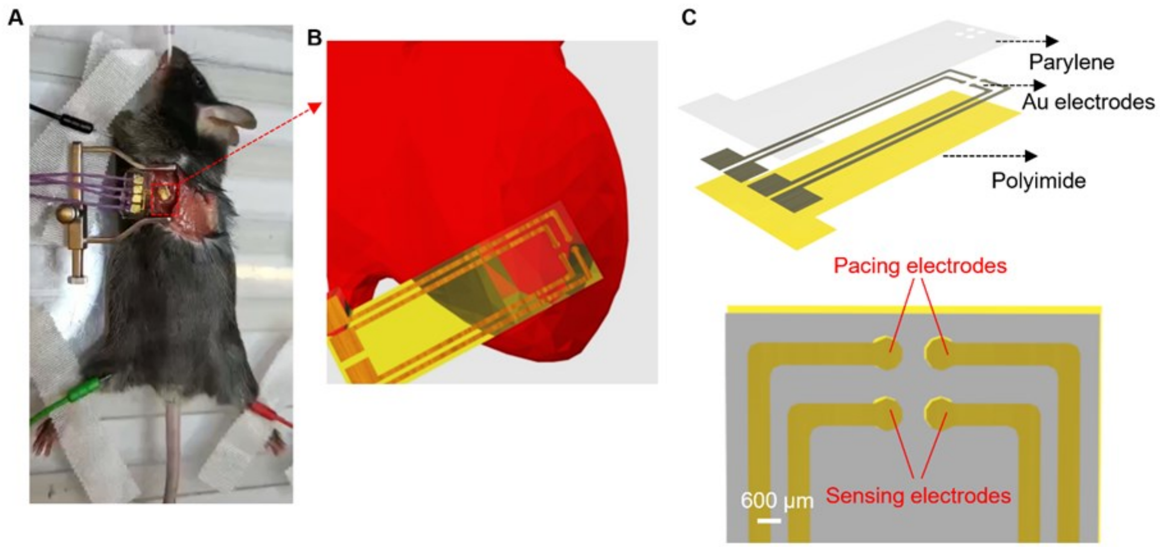

Fig. S47. Cardiac electrical stimulating in an *in vivo* mouse model. (**A**) Flexible electrical stimulation (E-stim) electrodes implantation. (**B**) Schematic illustration showing the cardiac patch containing a pair of E-stim electrodes and a pair of sensing electrodes mounted on the mouse left ventricle (LV). (**C**) The cardiac patch is composed of an encapsulation layer parylene (thickness ~2 μm), E-stim and sensing electrodes of Au (thickness ~200 nm) and a substrate layer of polyimide (thickness ~10 μm).

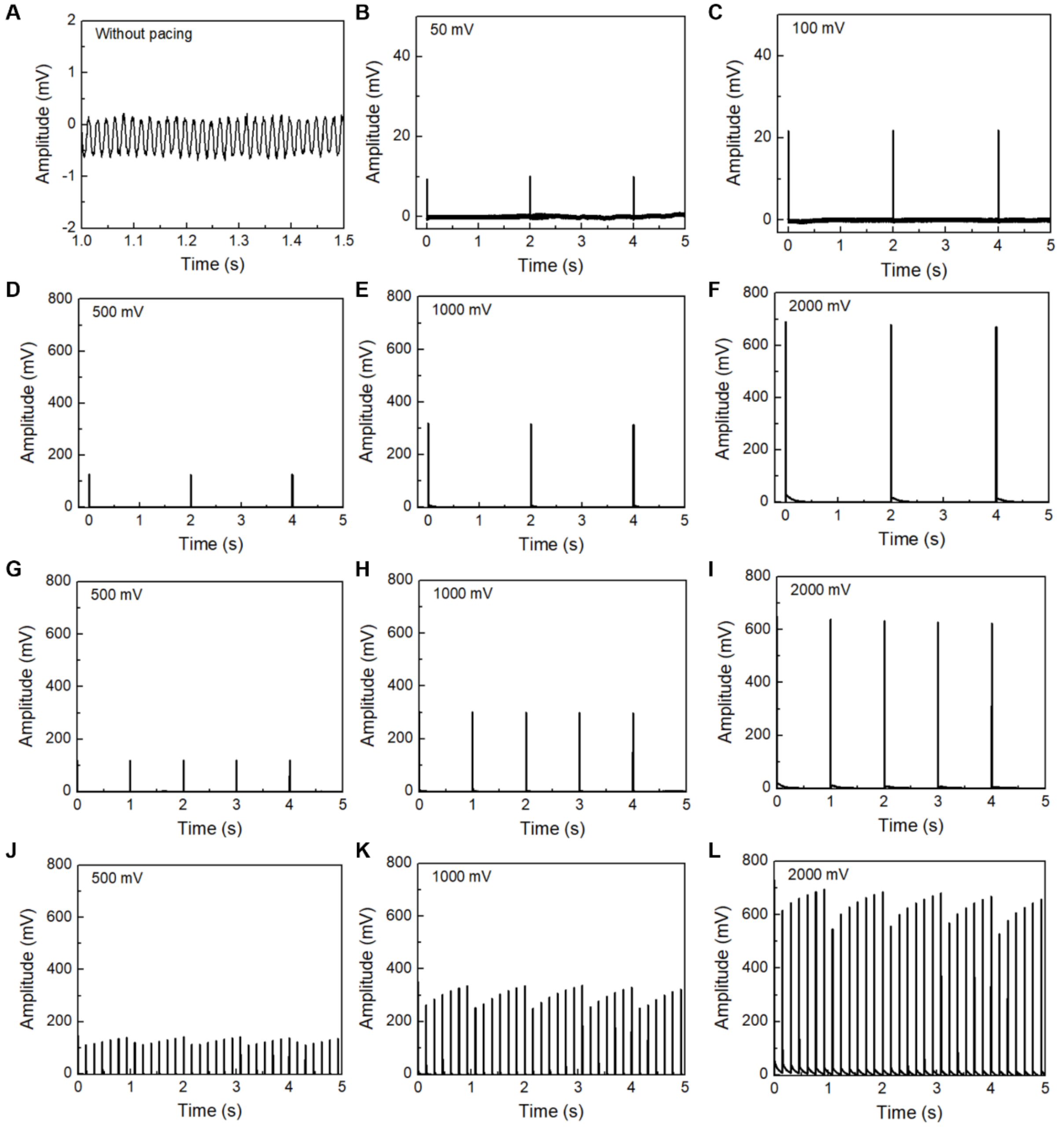

Fig. S48. Electrical activity recorded *in vivo* from the mouse heart with sensing-stimulating bimodal design. (**A**)The electrical activity recorded with a pair of sensing electrode before electrical stimulation (E-stim). (**B-L**) Representative voltage traces of the cardiac electrical activity during E-stim with various parameters. (**B**) 50 mV voltage with 1ms width at 0.5 Hz. (**C**) 100 mV voltage with 1ms width at 0.5 Hz. (**D**) 500 mV voltage with 1ms width at 0.5 Hz. (**E**) 1000 mV voltage with 1ms width at 0.5 Hz. (**F**) 2000 mV voltage with 1ms width at 0.5 Hz. (**G**) 500 mV voltage with 1ms width at 1.0 Hz. (**H**) 1000 mV voltage with 1ms width at 1.0 Hz. (**I**) 2000 mV voltage with 1ms width at 1.0 Hz. (**J**) 500 mV voltage with 1ms width at 6.5 Hz. (**K**) 1000 mV voltage with 1ms width at 6.5 Hz. (**L**) 2000 mV voltage with 1ms width at 6.5 Hz.

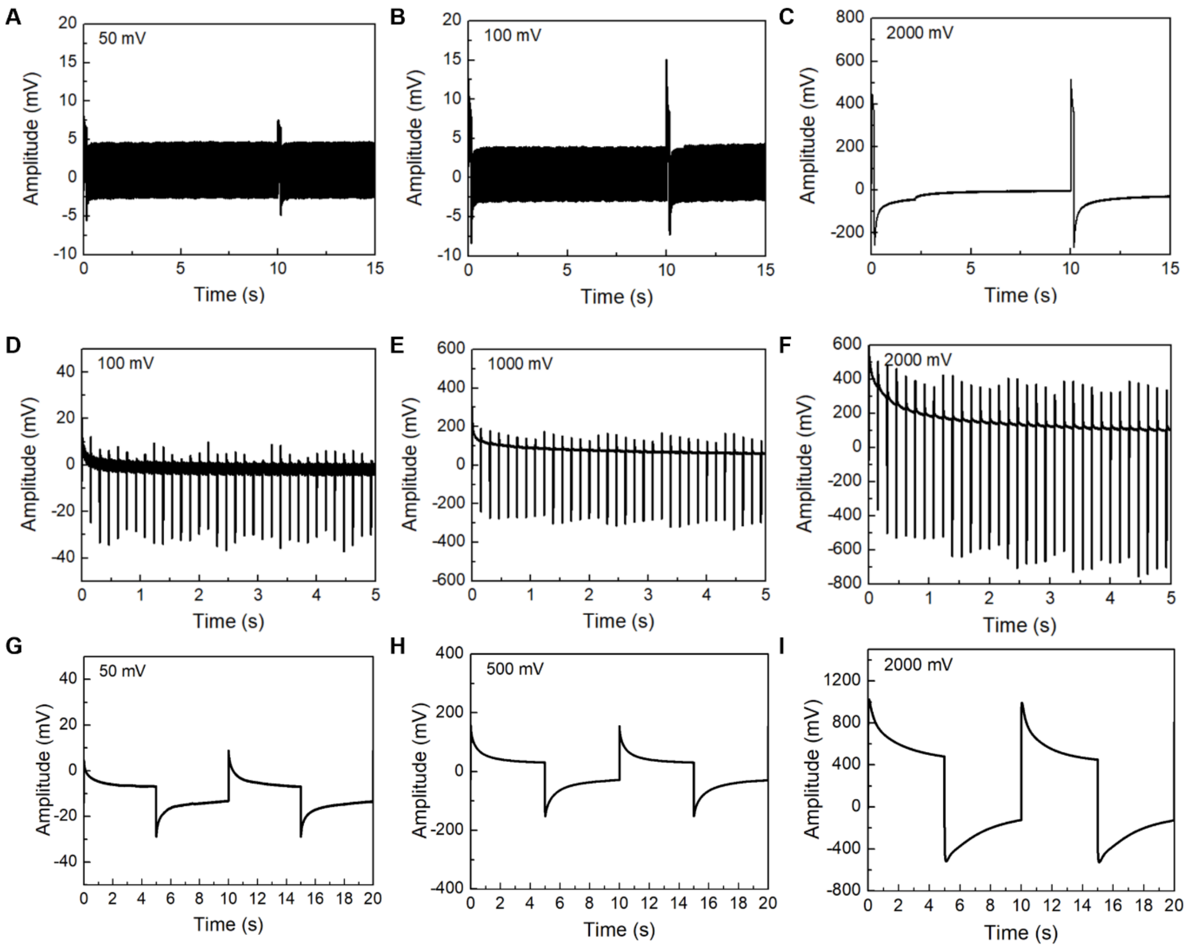

Fig. S49. Representative voltage traces of the cardiac electrical activity during E-stim with various parameters recorded with a pair of sensing electrode. (**A**) 50 mV voltage with 150 ms width at 0.1 Hz. (**B**) 100 mV voltage with 150 ms width at 0.1 Hz. (**C**) 2000 mV voltage with 150 ms width at 0.1 Hz. (**D**) 100 mV voltage with 150 ms width at 6.5 Hz. (**E**) 1000 mV voltage with 150 ms width at 6.5 Hz. (**F**) 2000 mV voltage with 150 ms width at 6.5 Hz. (**G**) 50 mV voltage with 5 s width at 0.1 Hz. (**H**) 500 mV voltage with 5 s width at 0.1 Hz. (**I**) 2000 mV voltage with 5 s width at 0.1 Hz.

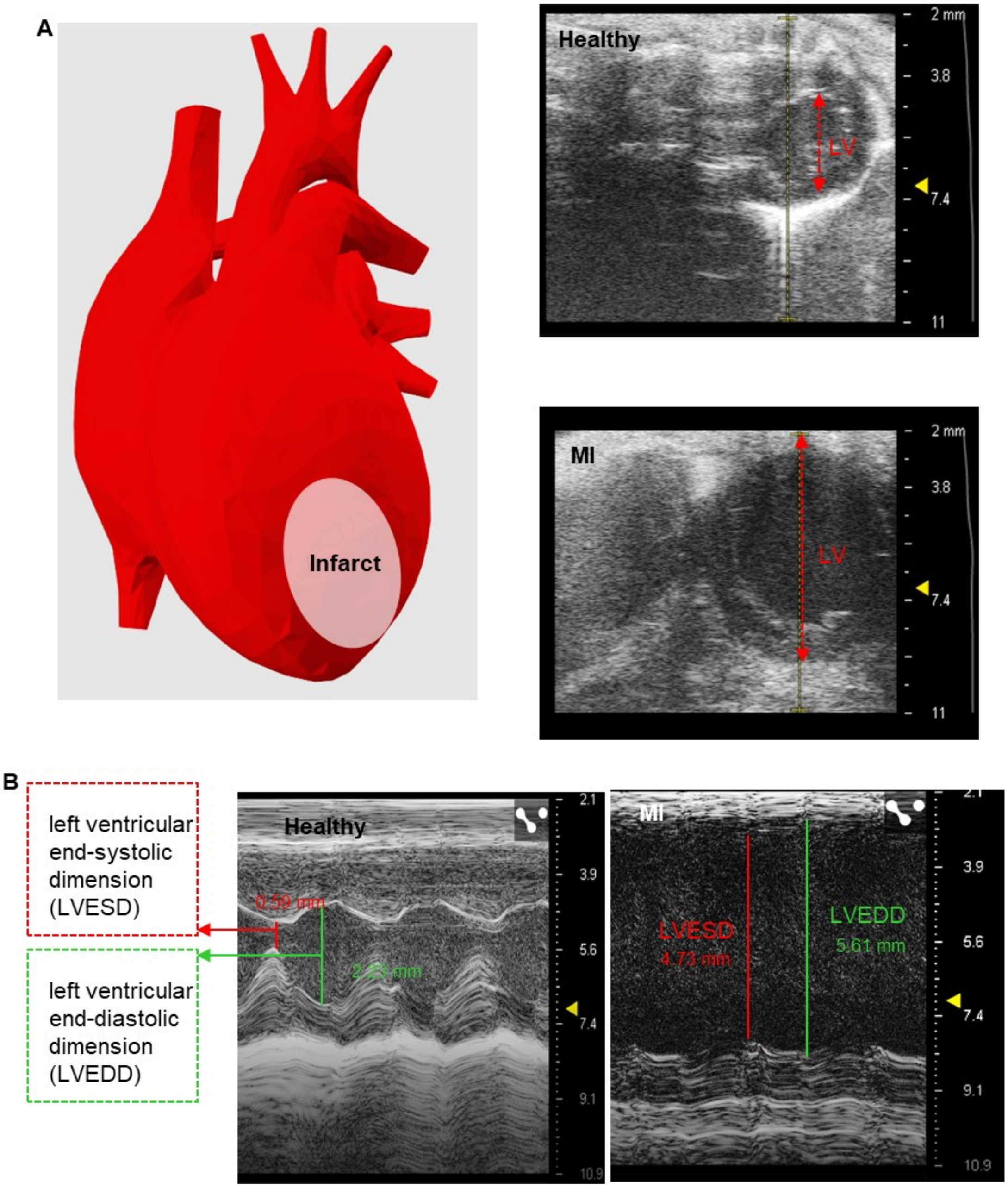

Fig. S50. Echocardiographic evaluation of left ventricular function. (**A**) Left: Schematic illustration of a heart after myocardial infarction. Here, the left coronary artery (LCA) of a living mouse is permanently ligated to cause the MI model. The infarcted area exhibits pale color since the myocardium begin die due to the lack of blood supply. Right: Corresponding B-mode echocardiographic images from a healthy heart and post-MI heart, respectively. (**B**) M-mode echocardiographic images from a healthy and post-MI heart.

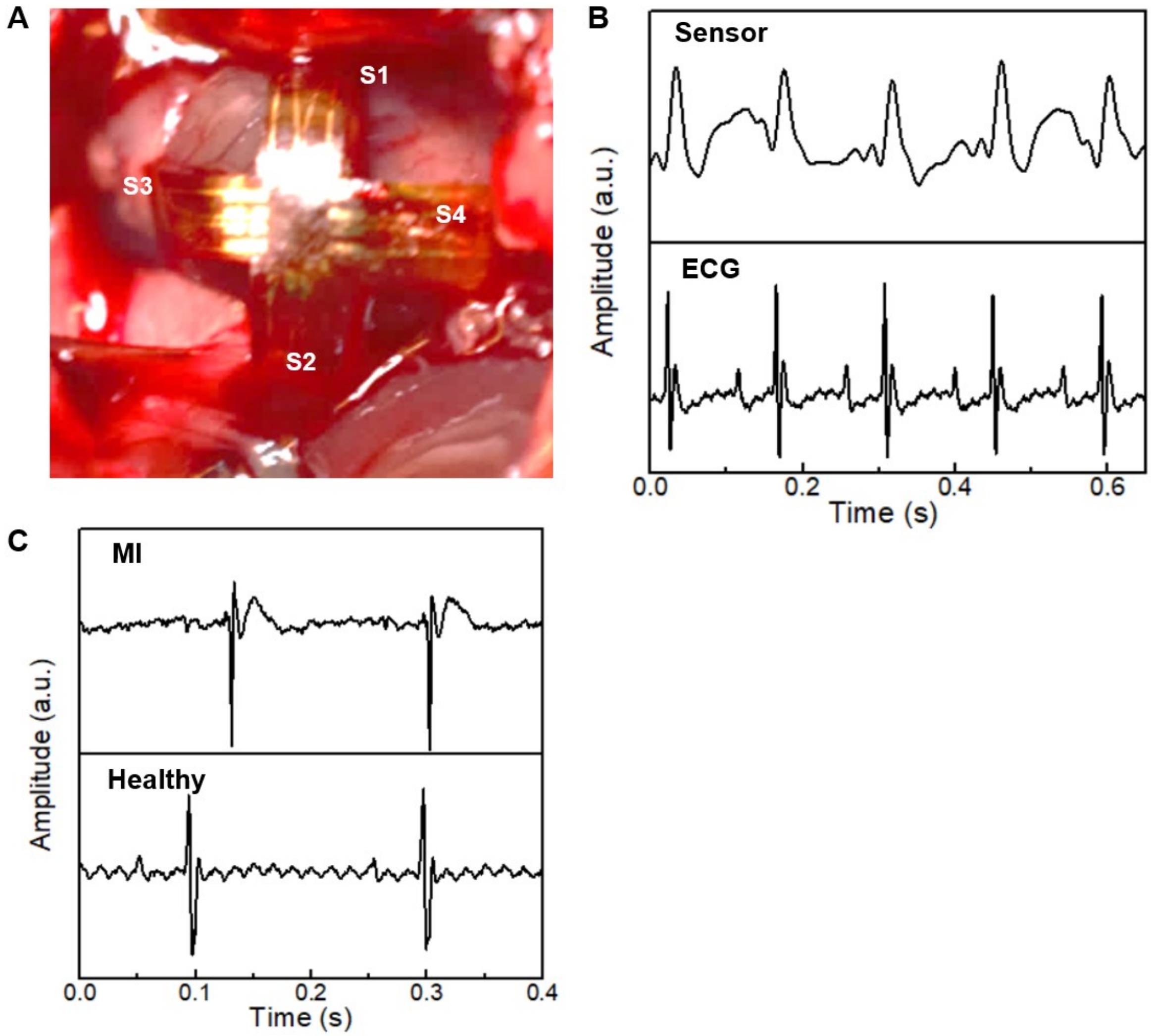

Fig. S51. A soft robotic thera-gripper with four sensing arms for monitoring cardiac contractility. (**A**) Optical image showing the thera-gripper grasps a mouse heart with four strain sensors (labeled as S1, S2, S3, and S4) engaged with different heart chambers for monitoring local cardiac contraction. (**B**) Responses of an Au strain sensor under representative physiological conditions, compared with simultaneous ECG recordings. (**C**) Measured ECG signals between a normal heart and a post-MI heart using the robotic gripper

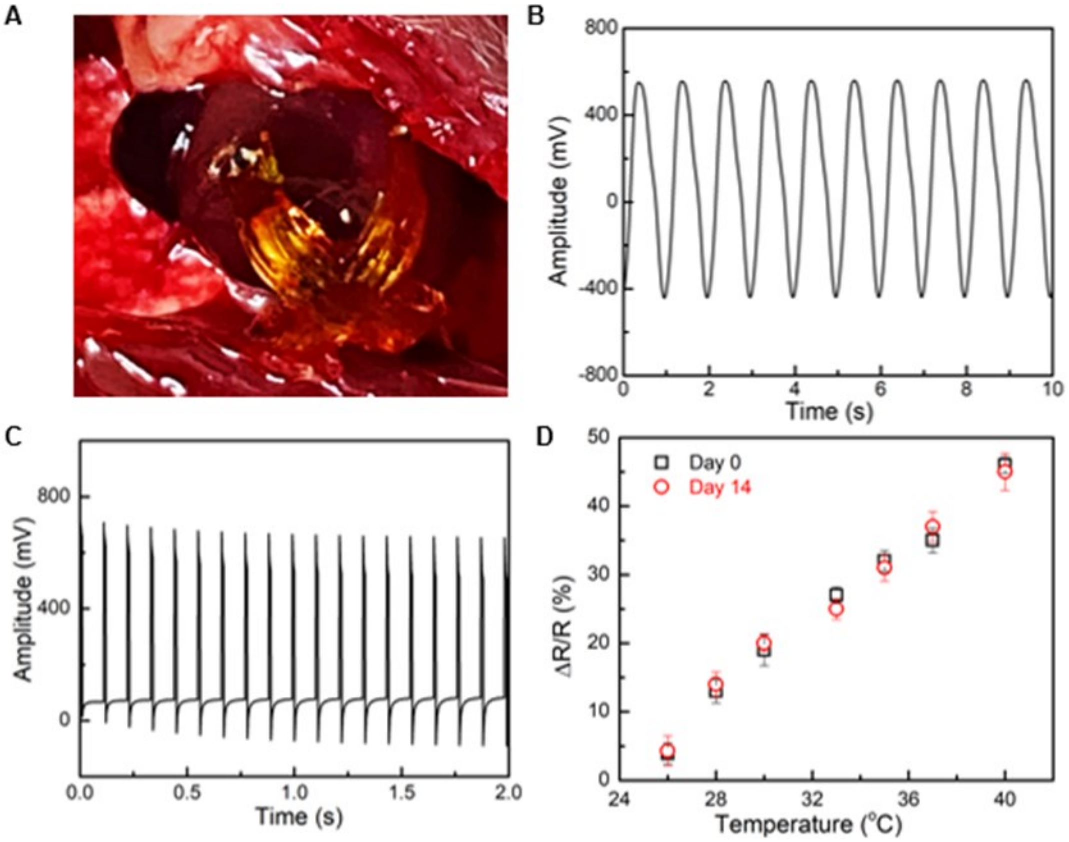

Fig. 52. Overview of device performance after two-week implantation. (**A**) Visualization of the device accurately positioned on the mouse heart, indicating its stability post two-week implantation. (**B&C**) Voltage response traces from the E-stim electrodes embedded in the implanted device, demonstrating its operational integrity over the two-week period. Here, (**B**) shows a sine wave configuration at a frequency of 1 Hz and amplitude of 1 V, while (**C**) depicts pulse modulation with a height of 500 mV, pulse with of 0.01 s, and a maximum repeating rate of 1Hz. (**D**) Comparative analysis of the resistive response from the thermal sensor before and after the two-week implantation period, illustrating the device's consistent performance and sensor integrity over time.

Supplementary movie 1: Soft robot with a nature-inspired starfish design

Supplementary movie 2: Shape transformation of soft robots with various configurations

Supplementary movie 3: Soft robot with a helical structure biomimicking a chiral seedpod

Supplementary movie 4: The shape transformation of a soft robotic pill

Supplementary movie 5: A three-arm soft robotic gripper via sequentially programming input power

Supplementary movie 6: A four-arm soft robotic gripper via simultaneously programming input power
